# H_2_ roaming chemistry and the formation of H_3_^+^ from organic molecules in strong laser fields

**DOI:** 10.1038/s41467-018-07577-0

**Published:** 2018-12-05

**Authors:** Nagitha Ekanayake, Travis Severt, Muath Nairat, Nicholas P. Weingartz, Benjamin M. Farris, Balram Kaderiya, Peyman Feizollah, Bethany Jochim, Farzaneh Ziaee, Kurtis Borne, Kanaka Raju P., Kevin D. Carnes, Daniel Rolles, Artem Rudenko, Benjamin G. Levine, James E. Jackson, Itzik Ben-Itzhak, Marcos Dantus

**Affiliations:** 1 Department of Chemistry, Michigan State University, East Lansing, MI 48824 USA; 2 J. R. Macdonald Laboratory, Physics Department, Kansas State University, Manhattan, KS 66506 USA; 3 Department of Physics and Astronomy, Michigan State University, East Lansing, MI 48824 USA

## Abstract

Roaming mechanisms, involving the brief generation of a neutral atom or molecule that stays in the vicinity before reacting with the remaining atoms of the precursor, are providing valuable insights into previously unexplained chemical reactions. Here, the mechanistic details and femtosecond time-resolved dynamics of H_3_^+^ formation from a series of alcohols with varying primary carbon chain lengths are obtained through a combination of strong-field laser excitation studies and ab initio molecular dynamics calculations. For small alcohols, four distinct pathways involving hydrogen migration and H_2_ roaming prior to H_3_^+^ formation are uncovered. Despite the increased number of hydrogens and possible combinations leading to H_3_^+^ formation, the yield decreases as the carbon chain length increases. The fundamental mechanistic findings presented here explore the formation of H_3_^+^, the most important ion in interstellar chemistry, through H_2_ roaming occurring in ionic species.

## Abstract (web summary)

H_2_ roaming is associated with H_3_^+^ formation when certain organic molecules are exposed to strong laser fields. Here, the mechanistic details and time-resolved dynamics of H_3_^+^ formation from a series of alcohols were obtained and found that the product yield decreases as the carbon chain length increases.

## Introduction

As one of the most abundant, yet simplest, triatomic cations in the universe, the trihydrogen cation^1,2^ (H_3_^+^) plays a vital role in interstellar gas-phase chemistry by facilitating the formation of molecules such as water and hydrocarbons. Clues regarding the fundamental dynamics and mechanisms of these chemical processes may be obtained from laser-induced dissociation processes producing H_3_^+^. The production of H_3_^+^ from various organic molecules following excitation with intense femtosecond laser pulses has been reported previously^3–13^. However, the exact mechanism(s), timescale(s), and yield(s) for this reaction have remained a mystery. In a recent study^14^, we provided experimental and theoretical evidence for the existence of two reaction pathways for the formation of H_3_^+^ from methanol under strong-field ionization. In brief, both reaction pathways are initiated by the ultrafast double ionization of the parent molecule and proceed through prompt formation of a roaming neutral H_2_ moiety from the methyl site. By roaming, here we imply that a neutral fragment explores relatively flat regions of the potential energy surface far from the minimum energy path^15–19^. In doubly ionized methanol, the roaming H_2_ fragment abstracts a third proton from the methyl carbon or from the hydroxyl oxygen leading to the formation of H_3_^+^. Experimental findings for methanol and its isotopologues showed that the proton transfer is faster from carbon than from oxygen. In both these H_3_^+^ formation pathways, the multiple bond cleavage and bond formation processes, and the roaming of the neutral H_2_ moiety, all occur within a 100–250 fs timescale in our experiments. This recent finding, associating neutral H_2_ roaming with the formation of H_3_^+^ under strong-field excitation, inspired a series of additional experiments aimed at elucidating aspects of this novel chemical reaction mechanism.

The recent recognition of roaming mechanisms has deepened our understanding of certain exotic chemical reactions^15–19^. Roaming has been widely observed in highly excited polyatomic molecules, for which photodissociation proceeds through trajectories other than the minimum energy pathway. Typically, roaming takes place on a flat region of the potential energy surface, thus allowing nascent reaction products to remain near each other long enough for further reactions to occur. Of particular interest to the work presented, recent studies have focused on roaming processes in small organic molecules such as acetaldehyde^20–22^, acetone^23^, methyl formate^24–26^, and propane^27^. In most cases, the roaming pathway contributes a small fraction of the total yield. However, certain photodissociation pathways, such as the visible light-induced NO_3_ → NO + O_2_ decomposition reaction, occur solely via a roaming mechanism^28,29^. Similarly, the roaming of a neutral hydrogen molecule is essential for H_3_^+^ formation from the methanol dication^14^.

While roaming mechanisms have been well established in neutral molecules, the same cannot be said about ionic species. Outside of our work, to the best of our knowledge there is only one theoretical prediction of H_2_ roaming in the dissociative ionization of allene^30^. A theoretical analysis on the H_3_^+^ formation reaction from ethane identified a transition state with a H_2_ molecule attached to a C_2_H_4_^2+^ ion^8^. However, in that study, no evidence was provided confirming H_2_ roaming during the dissociation of the ethane dication. Based on the above background, together with our previous work^14^, one might speculate that all H_3_^+^ formation pathways originating from organic molecules require neutral H_2_ roaming, regardless of whether such mechanisms are initiated by charged particles or intense femtosecond laser fields. While scientifically confirming or refuting the validity of such a general statement is beyond the scope of this work, a systematic study of H_3_^+^ formation reactions on a family of molecules can provide valuable information about this relatively unknown H_2_ roaming mechanism in ionic species. Our work on the simplest alcohol cation^14^, and the follow-up experimental and theoretical work presented here on a series of alcohols, constitute most of what is known about H_2_ roaming and H-migration mechanisms occurring in ionic species. Here the roaming H_2_ molecule acts as a Brønsted–Lowry base,^31,32^ accepting a proton from the highly acidic doubly-charged fragment ion.

As the initiator of many interstellar chemical reactions, H_3_^+^ is a catalyst for the formation of dense molecular clouds containing complex organic molecules^33,34^. The formation reaction^35^, existence in interstellar space^36,37^, and spectroscopic properties^38^ of the H_3_^+^ ion, as well as its importance in the ion chemistry of interstellar molecular clouds are well documented^39–41^. In interstellar media, an environment rich in molecular hydrogen, protonation of molecular hydrogen is initiated by cosmic radiation. As proposed by Hogness and Lunn^35^, a bimolecular reaction involving neutral and singly ionized hydrogen molecules yields H_3_^+^, i.e., H_2_ + H_2_^+^ → H_3_^+^ + H. It is worth noting here that the proton abstraction by H_2_ observed in methanol resembles the Hogness and Lunn reaction leading to the formation of H_3_^+^. In addition to its formation through femtosecond laser excitation, H_3_^+^ has been observed from certain organic molecules via electron impact^42–44^, proton impact^45–47^, and highly-charged ion collision^48,49^ under laboratory conditions. Most of what is known about H_3_^+^ chemistry comes from reactive scattering experiments and from ion-neutral reactions in flow drift tubes. These measurements, full collisions, provide reaction cross sections and in some cases angle-resolved product state distributions^50–52^. Unimolecular photodissociation reactions, half collisions^53,54^, proceed from a well-defined geometry and can be studied with femtosecond time resolution^55^. Here we apply the concept of half-collision to learn about the femtosecond dynamics of reactions involving H_3_^+^ produced from alcohols^56^ and their importance in astrochemistry in the formation of larger complex molecules through protonation^57,58^. Our study helps reveal dynamics and mechanistic details that are not measurable in reactive ion-neutral scattering studies. Furthermore, our findings are relevant to chemistry initiated by cosmic radiation including photons and electrons with energies in the 30–100 eV range. Given the abundance of hydrogen in organic compounds, solvents, and fuels, the neutral hydrogen roaming chemical reactions discussed in this work may be relevant to condensed-phase chemical reactions involving superacids^59^, soot formation in combustion chemistry^60^, charged particle-impact-induced chemical reactions^61^, and gas-phase acid/base reactions including those that formed the first organic compounds in the universe^2^.

**Fig. 1:**
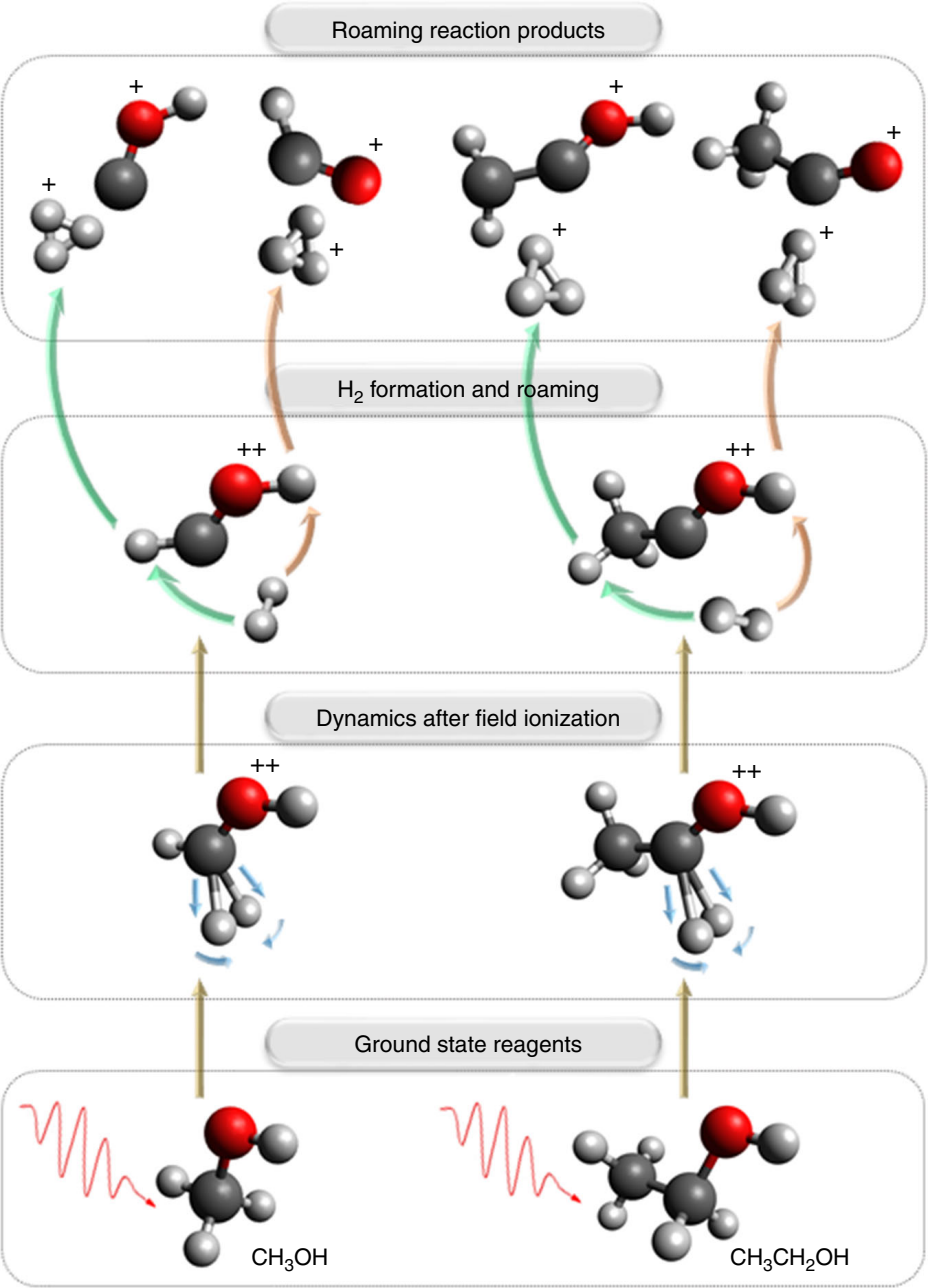
Primary H_3_^+^ formation pathways from methanol (CH_3_OH) and ethanol (CH_3_CH_2_OH). Formation occurs via the neutral H_2_ roaming mechanism under strong-field laser ionization. In both molecules, the carbon atom attached to the hydroxyl functional group is referred to as the α-carbon and the corresponding hydrogen atoms are referred to as α-hydrogens. In the case of ethanol, the terminal carbon atom and hydrogen atoms are referred to as the β-carbon and the β-hydrogen atoms, respectively

In this article, we examine the involvement of H_2_ roaming mechanisms in ionic species in the formation of H_3_^+^ from a series of alcohols under strong-field excitation and investigate the effect that longer carbon chains have on the yield of H_3_^+^. The formation pathways of H_3_^+^ via the roaming H_2_ mechanism for methanol and ethanol, which are triggered by the strong-field double ionization of the corresponding parent molecule, are shown in Fig. 1. When comparing the doubly-charged structures to their neutral counterparts, one can clearly see the elongation of C–H bonds and the narrowing of the H–C–H angle on the α-carbon atom. Based on our previous study^14^, we consider that these intramolecular changes are the primary motions leading to the formation of neutral H_2_ and eventually of H_3_^+^. Here we evaluate the validity of this mechanism and the influence of alkyl-chain length by extending our work to a series of primary alcohol molecules: methanol (CH_3_OH), ethanol (CH_3_CH_2_OH), and 1-propanol (CH_3_CH_2_CH_2_OH). Findings for a secondary alcohol, 2-propanol (CH_3_CH(OH)CH_3_), and a tertiary alcohol, *tert*-butanol ((CH_3_)_3_COH), species that cannot react as proposed in Fig. 1, are then compared. Through in-depth experimental analysis of product yields and timescales of formation at a peak laser intensity of 2.0 × 10^14^ W cm^−2^, we show that H_3_^+^ yield decreases as the primary carbon chain length increases. Furthermore, we reveal additional formation mechanisms available for the production of H_3_^+^ in small alcohols, particularly from ethanol.

## Results

### Experimental H_3_^+^ yields

In order to compare H_3_^+^ production from different alcohols upon ultrafast double ionization, we employed two distinct experimental setups and analysis methods. Using time-of-flight (TOF) mass spectrometry, we are able to compare the total yield of H_3_^+^ (i.e. the integral over that peak) for each of the different alcohols. Complete TOF mass spectra (TOF-MS) for methanol, ethanol, 1-propanol, 2-propanol, and *tert*-butanol are given as Supplementary Information Figs. 1‒5. For these measurements we carefully controlled the laser excitation and the target density. In order to quantify the H_3_^+^ branching ratio following double ionization, we carried out coincidence TOF (CTOF) measurements where we directly counted the number of events leading to the H_3_^+^ formation relative to all dications produced. Further information regarding experimental techniques, setups, and parameter settings can be found in the Methods section.

**Fig. 2:**
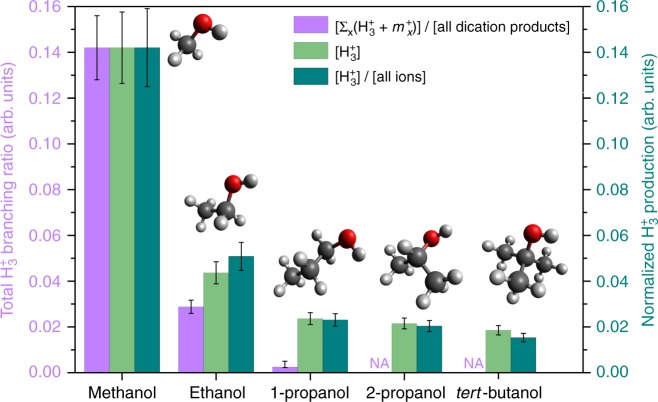
H_3_^+^ production from a series of alcohols. Total H_3_^+^ branching ratios ([Σ_*x*_(H_3_^+^ + *m*_*x*_^+^)]/[all dication products]) together with normalized H_3_^+^ ([H_3_^+^]), and fractional H_3_^+^ ([H_3_^+^]/[all ions]) production from dissociative ionization of methanol, ethanol, 1-propanol, and 2-propanol together with *tert*-butanol in a linearly polarized laser field with a peak intensity of 2.0 × 10^14^ W cm^−2^. The [H_3_^+^] and [H_3_^+^]/[all ions] yields were obtained through the TOF technique, and each of them is normalized with respect to the corresponding branching ratio of methanol, [(H_3_^+^ + HCO^+^)]/[all dication products], determined by the CTOF method. Due to the complexity of quantitative analysis (see text for details), CTOF measurements were not performed for large molecules (2-propanol and *tert*-butanol) and indicated by “NA” at the corresponding positions in the figure. Data are provided as Supplementary Table 1

Quantifying the CTOF branching ratio requires consideration of every coincidence ion pair associated with the production of H_3_^+^. Specifically, we determined the sum of all measured ion pairs containing H_3_^+^ (i.e. H_3_^+^ + *m*_*X*_^+^) divided by the sum of all single ions and ion pairs originating from the parent dication (i.e. all dication products). Extraction of the pair coincidences is illustrated in Supplementary Fig. 6. The analytical expression for the CTOF branching ratio is given in the Methods: Experimental setup section (Eq. 2). As explained in Supplementary Note 1, this method has the advantage that it allows a direct comparison of branching ratios among different molecules. While this accounting is relatively easy to do for methanol, it is challenging for ethanol, and very complicated and time consuming for larger molecules. The results from our measurements on a series of alcohols are presented in Fig. 2. The first column (purple) corresponds to the CTOF-determined branching ratio, the second column (light green) corresponds to the integrated H_3_^+^ TOF yield ([H_3_^+^]), and the third column (dark green) corresponds to the H_3_^+^ TOF yield normalized by the total number of ions detected. For comparison, the TOF measurements have been normalized to the measured H_3_^+^ branching ratio of methanol obtained by CTOF measurements.

What is immediately visible in Fig. 2 is the reduction in the H_3_^+^ formation with increasing carbon chain length, regardless of the measurement technique or the normalization method. Formation of H_3_^+^ is most prominent from methanol, even though it has fewer hydrogen atoms per molecule than the other molecules of interest. In the case of ethanol, the production of H_3_^+^ is smaller by a factor of about 5 or 3 (for the CTOF and TOF data, respectively) compared to that of methanol. This observation seems counterintuitive; ethanol contains 50% more hydrogen atoms than methanol, suggesting that additional H_3_^+^ formation pathways might have been expected, resulting in a higher H_3_^+^ yield. To our surprise, upon further lengthening the carbon chain, i.e. in the case of 1-propanol, the total H_3_^+^ production drops by an additional factor of 11 or 2 (for CTOF and TOF measurements, respectively) compared to ethanol.

When expressed as a fractional yield, i.e. [H_3_^+^]/[all ions] (dark green column in Fig. 2), the TOF results follow a similar trend as observed with total H_3_^+^ branching ratios for the smaller three molecules for which CTOF measurements are not too demanding. Though the fractional yield is proportional to the branching ratio for H_3_^+^ production, a derivation detailed in the Supplementary Note 1 indicates that the proportionality coefficient depends on the ratio of single to double ionization probabilities, denoted by *σ*_1_ and *σ*_2_, respectively. Explicitly this relation is given by

1 $$\mathop {\sum}\limits_j {F_2\left( {3,j} \right)} \simeq \left( {\frac{{\sigma _1}}{{\sigma _2}} + 1} \right)\frac{{M\left( 3 \right)}}{\mathop {\sum}\limits_k {M\left( k \right)}}{,}$$

where *F*_2_(3, *j*) is the branching ratio of H_3_^+^ + *m*_*j*_^+^ and *M*(*i*) is the number of counts measured in a specific TOF peak associated with mass *m*_*j*_ (assuming singly-charged for simplicity). In spite of the additional dependence on the ionization probabilities, the trend evaluated using the fractional H_3_^+^ yield is in reasonable agreement with the branching ratios evaluated directly from the CTOF measurements. This suggests that the *σ*_1_ to *σ*_2_ ratio, which can vary significantly from one molecule to another, varies slowly for the group of molecules in our study. The most likely explanation is that double ionization occurs predominantly from the oxygen atom in the hydroxyl group that is common in all these molecules.

Through our analysis, we learn that the TOF fractional H_3_^+^ production allows the discovery of H_3_^+^ formation trends, and that even a direct comparison of the measured TOF H_3_^+^ yield is consistent with the more complex and in-depth analysis-dependent CTOF method as long as one can maintain the experimental conditions between measurements on different molecules under tight control and the single to double ionization probability does not change significantly between molecules. It is worth noting here that the discrepancy in the CTOF and TOF data for 1-propanol could be attributed to the production of some H_3_^+^ from the mono-cation of 1-propanol (a more detailed error analysis is provided in Supplementary Note 2 and Supplementary Figs. 7–9). Having established the qualitative trends of both methods, and calibrated the TOF data with the best numbers obtained by CTOF on methanol, we are able to evaluate trends among the larger alcohols.

In Fig. 2, we observe that the integrated H_3_^+^ yield as well as the fractional H_3_^+^ yield from 1-propanol and 2-propanol, which have the same number of hydrogen atoms (and, most likely, have similar photoionization rates), is comparable within the measurement error. Clearly, the arrangement of hydrogen atoms within the molecules is significantly different; however, 2-propanol possesses a single α-hydrogen atom, which plays a key role in H_3_^+^ formation as we discuss in a later section of this work. In the case of *tert*-butanol, which has three terminal methyl groups and no α-hydrogen atom in its structure, we expected a reduction in the H_3_^+^ yield. However, no such reduction is observed. The observation of H_3_^+^ formation from *tert*-butanol implies that an H_3_^+^ formation mechanism exists that primarily involves hydrogen atoms from terminal methyl groups, without an involvement of α-hydrogen atoms. This observation is in agreement with the formation of H_3_^+^ from acetone^14^, in which hydrogen migration is not favorable and H_3_^+^ is solely produced from terminal methyl groups.

### Electronic structure calculations

Ab initio electronic structure calculations play an important role in further explaining the observed yields and branching ratios. Further information regarding electronic structure calculations is provided in the Methods: Ab initio calculations and simulations section and in Supplementary Note 3. In methanol, upon instantaneous double ionization of the parent molecule, the positive charges that build up on the oxygen atom draw electron density from the methyl group, thus decreasing the electron density on the hydrogen atoms, as shown in the calculated Mulliken population analysis for the neutral and doubly-charged molecules (Supplementary Fig. 11). This results in weakening of the C–H bonds, causing elongation and favoring detachment of H_2_ from the parent C atom (Supplementary Fig. 10), an essential step in initiating the roaming mechanism leading towards the formation of H_3_^+^. In the case of ethanol, the depletion of electron density on the α-carbon and the α-hydrogen atoms is partially compensated by the terminal methyl group. Therefore, the α-hydrogen atoms are less positively charged in the ethanol dication than in the methanol dication, as evident in the Mulliken population analysis of doubly-charged ethanol (Supplementary Fig. 11). One can also see that the positive charge on the β-carbon hydrogen atoms is smaller than the α-carbon ones; we therefore surmise that the β-carbon C–H bonds are less likely to favor H_2_ detachment, resulting in a reduced yield of H_3_^+^. The further reduction in H_3_^+^ yield found for 1-propanol can be attributed to the fact that the electronic induction from the terminal ethyl group is higher than that of the methyl group (Supplementary Fig. 11).

### New H_3_^+^ formation pathways

While Fig. 1 shows the primary mechanism for H_3_^+^ formation, the trends observed in Fig. 2 for the different alcohols indicate that other formation pathways exist. Here we address the different mechanisms for H_3_^+^ formation available to alcohols, focusing initially on the two-body fragmentation of ethanol. The complexity arising from having a larger number of hydrogen atoms is addressed by the judicious selection of partially deuterated ethanol isotopologues, which allows us to identify and clearly distinguish several different H_3_^+^ formation pathways.

**Fig. 3:**
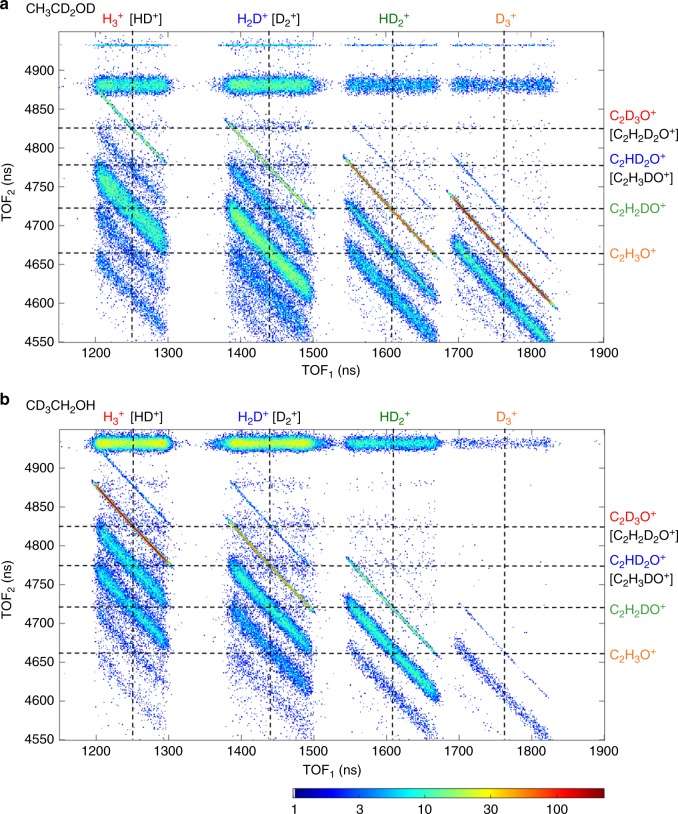
H_3_^+^ formation from ethanol. Truncated coincidence time-of-flight maps focused only on H_3_^+^ production in two-body channels from dissociative ionization of **a** CH_3_CD_2_OD and **b** CD_3_CH_2_OH in a linearly polarized laser pulse centered about 790 nm, 23-fs long with a peak intensity of 3.0 × 10^14^ W cm^−2^. The labeled dashed lines indicate the two-body breakup ion pairs related to H_3_^+^ formation from the ethanol dication. The logarithmic color scale depicts the number of ion pairs recorded

Figure 3 presents CTOF spectra from dissociative ionization of (a) CH_3_CD_2_OD and (b) CD_3_CH_2_OH obtained at a peak laser intensity of 3.0 × 10^14^ W cm^−2^, in which four H_3_^+^ formation pathways were clearly identified. Each correlated pair of ions (two-body breakup channel) occurs as a narrow diagonal streak on the two-dimensional ion arrival time map as a result of momentum conservation, for example the ion pair D_3_^+^ and C_2_H_3_O^+^. Data from an ion pair, with the second fragment having a lower mass (lower in the column) is associated with three-body dissociation involving neutral H or H_2_, and because the neutral fragment carries some momentum, the diagonal streak is broadened. Data from an ion pair, with the second fragment having a higher mass (higher in the column), is associated with a ^13^C isotopic impurity in the sample (further details pertaining to interpreting the CTOF spectra can be found in Supplementary Information Note 4 and Supplementary Fig. 12). For simplicity, we focus our discussion only on two-body breakup channels leading to the formation of H_3_^+^, which are labeled on Fig. 3. In Fig. 3a, we observe a well-defined, strong coincidence channel that corresponds to the formation of D_3_^+^. As supported by ab initio simulations (described later), we consider that D_3_^+^ formation proceeds via dissociation of a neutral D_2_ moiety from two deuterium atoms bound to the α-carbon followed by roaming and abstraction of the third proton from the hydroxyl group. This pathway is quite similar to what we observed in methanol^14^, in which the neutral H_2_ formed from the methyl group abstracts the hydroxyl proton to form H_3_^+^. The next prominent channel in Fig. 3a corresponds to the formation of HD_2_^+^, likely resulting when a similar neutral D_2_ moiety abstracts a β-hydrogen atom from the terminal methyl group. However, in our ab initio simulations, we observe that a roaming H_2_ moiety can be formed from one α- and one β-hydrogen. Therefore, in the formation of HD_2_^+^, we cannot exclude the possibility of an α-deuterium and a β-hydrogen migrating (as an HD fragment) and abstracting the oxygen-bound deuterium. Unfortunately, due to *m*/*z* degeneracy, the remaining two channels shown in Fig. 3a, i.e. H_2_D^+^ (with D_2_^+^) and H_3_^+^ (with HD^+^), do not provide conclusive evidence for any further pathways. However, as shown in Fig. 3b, by using CD_3_CH_2_OH we can isolate two additional channels for H_3_^+^ formation from ethanol, primarily involving β-deuterium atoms. The HD_2_^+^ formation channel results from two β-deuterium atoms associating with a single α-hydrogen atom or the oxygen-bound hydrogen. The fourth pathway we identified from CD_3_CH_2_OH is D_3_^+^ formation, which only involves β-deuterium atoms from the terminal methyl group. As evident in the later described ab initio simulations, these latter two pathways are most likely initiated by the migration of an α-hydrogen to the terminal methyl site, which enables the ejection of β-hydrogens. Beyond the identification of the above four pathways, further analysis regarding the multiple H_3_^+^ formation mechanisms will be presented elsewhere, in order to maintain the focus of this Communication on comparisons found among different alcohol molecules.

Returning to Fig. 2, it is noteworthy that 1-propanol, 2-propanol, and *tert*-butanol have approximately the same H_3_^+^ yield, within experimental errors. For 1-propanol, one might expect H_3_^+^ formation paths similar to those described for ethanol, namely the primary pathway described in Fig. 1, and H-migration. However, for 2-propanol the primary mechanism as described in Fig. 1 is no longer available. Therefore, H_3_^+^ formation must follow migration of the α-hydrogen toward either of the methyl groups, or occur directly from hydrogens of the methyl groups. Perhaps having two terminal methyl groups instead of one compensates for the absence of two α-carbon bound hydrogen atoms. Direct formation of H_3_^+^ from a terminal methyl group seems to be the most probable mechanism available for *tert*-butanol, but having three such terminal methyl groups makes up for not having an H-migration pathway available.

### H_3_^+^ formation timescales

**Fig. 4:**
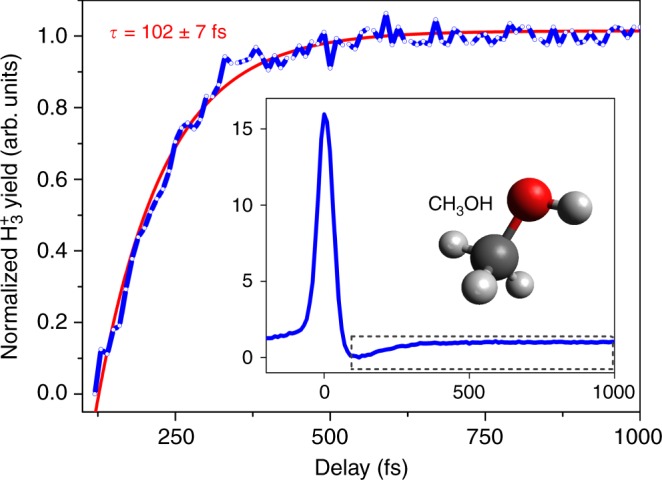
Pump-probe transient for H_3_^+^ production from methanol (CH_3_OH). Normalized H_3_^+^ yield (blue solid line) together with an exponential fit (red solid line) from dissociative ionization of methanol as a function of applied time delay between the pump and probe pulses. In the inset, the complete view of the normalized transient is shown where the dashed rectangle highlights the area of interest displayed in the main Figure. Normalization was performed such that the minimum value of the yield is 0 and the yield at large positive time delays (≥500 fs) is 1

The formation timescale of H_3_^+^ was experimentally obtained using a femtosecond pump-probe technique, which utilizes a strong pump pulse to generate the reaction precursor, the doubly-charged parent ion, and a weak probe pulse to interrupt the formation of H_3_^+^. Figure 4 presents the H_3_^+^ yield from methanol as a function of pump-probe time delay over a time period of 1.0 ps. A detailed description of time-dependent features of the complete transient (see Fig. 4 inset) can be found in our previous work^14^. In brief, a strong pump pulse creates the parent dication, which is then fragmented by the time-delayed weak probe pulse, thus preventing the formation of H_3_^+^. For short delay times, the probe arrives prior to the formation of H_3_^+^, the parent dication fragments and we observe a depletion in the H_3_^+^ yield. The incremental time delay between pump and probe pulses results in an exponential rise that tracks the H_3_^+^ formation time and reaches a plateau at long time delays > 500 fs. Using a mono-exponential fit given by *y* = *y*_0_ + *A* (1 − exp(−*t/τ*)), where *A* is the amplitude, *y*_0_ is the offset, and *τ* is the time constant, we extracted the formation time of H_3_^+^. Considering a 95% confidence level for the fit parameters, we observed a fast formation of H_3_^+^ from methanol, specifically *τ* = 102 ± 7 fs, which is in good agreement with our previous work^14^ (*τ* = 98 ± 4 fs). Furthermore, as observed in previously published ab initio molecular dynamics simulations^14^, the measured value is in good agreement with the H_3_^+^ formation time range of 50–150 fs for the mechanism involving the three hydrogen atoms from the methyl group.

**Fig. 5:**
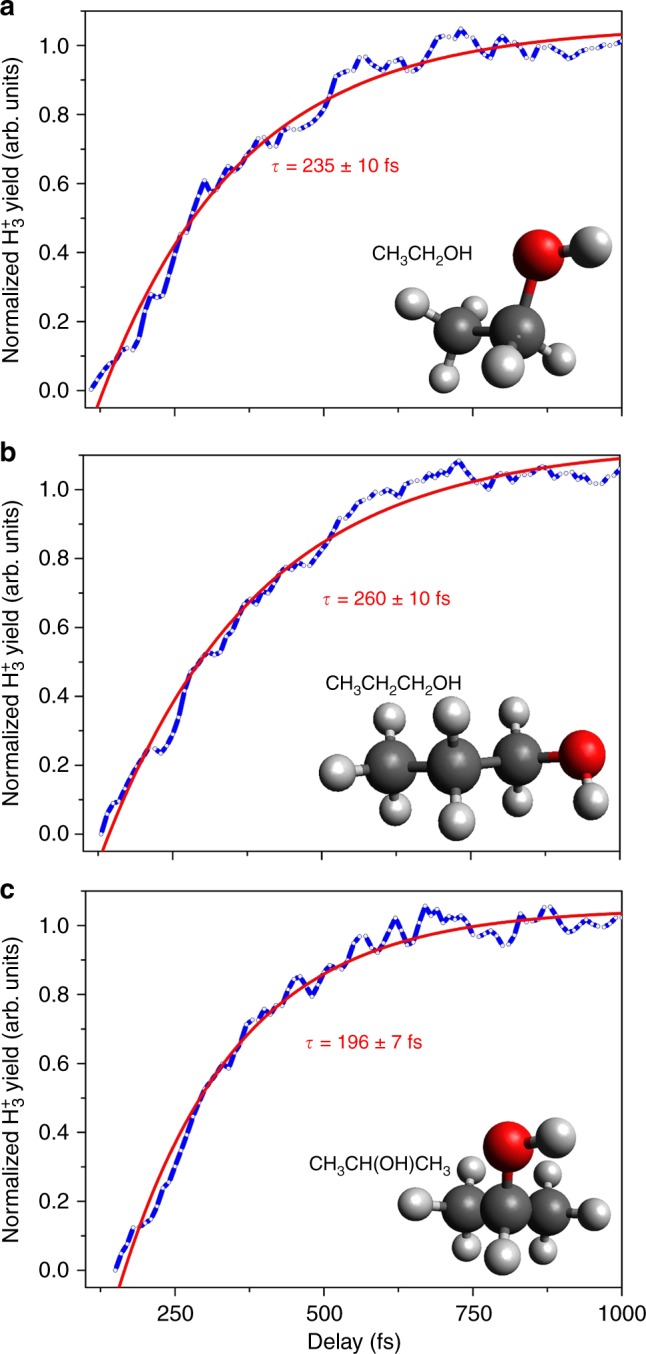
Pump-probe transient for H_3_^+^ production from alcohols. Normalized H_3_^+^ transients from dissociative ionization of different alcohols as a function of applied pump-probe delay. Shown in the figure (in blue solid lines) are **a** H_3_^+^ from ethanol, **b** H_3_^+^ from 1-propanol, and **c** H_3_^+^ from 2-propanol. Normalization was performed as described in Fig. 4 caption. Corresponding exponential fits are shown by red solid lines

Figure 5 presents the pump-probe transients of H_3_^+^ yields as a function of applied time delay over a time period of 1.0 ps for ethanol, 1-propanol, and 2-propanol. Subsequent to a similar exponential fit described previously, ethanol and 1-propanol exhibit formation times a factor of 2.3 ± 0.2 longer than for methanol, while the formation time for H_3_^+^ from 2-propanol only increased by a factor of 1.9. Our previous study^14^ found that the roaming H_2_ molecule abstracts the third proton from the same atom (carbon) faster (~100 fs) than from the adjacent atom (oxygen), due to the longer roaming time (and distance) of the neutral H_2_ moiety. With no third α-hydrogen atom available in ethanol or 1-propanol, this latter, slower H_3_^+^ formation channel is expected to dominate, as supported by our ab initio simulations. Here the roaming H_2_ molecule forms from the α-hydrogens and then abstracts the third hydrogen from the adjacent hydroxyl group. In both molecules, the H_3_^+^ formation from the terminal CH_3_ group is assumed to be negligible (as justified by the very weak D_3_^+^ + CH_2_OH^+^ two-body coincidence channel in Fig. 3b). Interestingly, 2-propanol exhibits a formation time for H_3_^+^ slower than the dominant path in methanol, but faster than those of ethanol and 1-propanol. Clearly, a distinct formation mechanism takes over when the terminal methyl hydrogens must be involved.

### Ab initio molecular dynamics simulations

An adequate first-principles molecular dynamics scheme for the fragmentation of ethanol requires a method that provides a balanced description across all potential closed and open shell fragments, such as the complete active space self-consistent field (CASSCF) method, which was implemented using an active space of 12 electrons in 12 orbitals. A summary of the final hydrogen dissociation products following ab initio molecular dynamics simulations of the photodissociation of doubly-ionized ethanol is reported in Table 1. We notice that the largest pathway is H^+^ formation, which is in agreement with the experimental yield as the H^+^ ion peak is the strongest. The H_2_ and H_2_^+^ channels were minimal compared to H^+^. H_3_^+^ formation was not observed in our CASSCF trajectories due to the limited sampling afforded by these high-level calculations and the low H_2_ formation yield.

**Table 1:** Percentage of hydrogen species (summed over all channels) ejected from doubly-charged ethanol that are observed using CASSCF and QCISD ab initio molecular dynamics simulations

	CASSCF % yield	QCISD % yield
H^+^ formation	38.6	30.1
H_2_^+^ formation	0.5	0
H_2_ formation	2.6	57.9
H_3_^+^ formation	0	0.2

Our main interest throughout the calculations is to understand and elucidate the H_3_^+^ formation mechanism in ethanol and whether it proceeds through formation of a roaming neutral H_2_ followed by abstraction of a proton to form H_3_^+^ as found in methanol^14^. In hopes of observing H_3_^+^ formation, we have also carried out molecular dynamics simulations with the electronic structure computed at the quadratic configuration interaction singles and doubles (QCISD) level, which can adequately describe closed shell pathways, such as H_2_ and H_3_^+^ formation. Though this method surely overestimates the probability of neutral H_2_ formation, it also likely provides a very accurate representation of the dynamics following the formation of neutral H_2_. To support this assessment, we have benchmarked the validity of QCISD for the formation of H_3_^+^ relative to our previous CASSCF calculations^14^ using methanol as shown in the Supplementary Note 5. In the benchmark study, we noticed that QCISD was incapable of predicting the formation of the open shell fragment H_2_^+^, and at the same time QCISD was biased toward H_2_ and H_3_^+^ formation as can be deduced from their higher yields compared to the CASSCF results. The observed probability of H_3_^+^ formation upon release of H_2_ was comparable at the CASSCF and QCISD levels, and the qualitative mechanism was observed to be similar regardless of method (Supplementary Table 2). This mechanism commences with the formation of a neutral H_2_ moiety from the α-hydrogen atoms that roams for a brief period until it abstracts a proton from the terminal methyl site or the hydroxyl site. Thus, QCISD is sufficient to understand the H_2_ formation pathways as well as H_3_^+^ production. Ethanol QCISD results are summarized in Table 1, which shows that H_2_ formation is relatively high through which H_3_^+^ formation is observed as well.

**Fig. 6:**
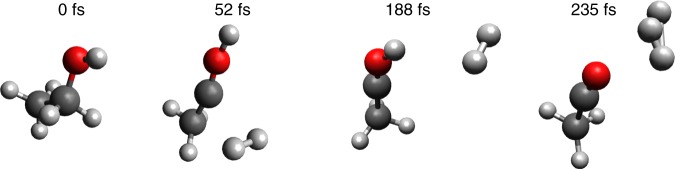
Molecular dynamics trajectory for H_3_^+^ formation. Snapshots at different times from a representative trajectory showing formation of H_3_^+^ from ethanol calculated using QCISD ab initio molecular dynamics. The complete trajectory is provided as Supplementary Video 1

Two videos of representative H_3_^+^-forming molecular dynamics trajectories are included in the online Supplementary Information (Supplementary Videos 1 and 2). These videos show that H_2_ forms from the two α-hydrogens, then roams and abstracts a proton from the hydroxyl group to form H_3_^+^ (snapshots of a trajectory are shown in Fig. 6). In our molecular dynamics simulations, all the observed H_3_^+^ trajectories (4 events out of 2000 trajectories) followed the same mechanism, in which a neutral roaming H_2_ formed from two α-hydrogens, then abstracts the hydroxyl hydrogen on timescales between 110 and 220 fs. This is in good agreement with our experimental observations for ethanol presented in a previous section of this study.

The molecular dynamics trajectories were sampled from the ground state minima, which correspond to geometries far from minima on the doubly-charged potential energy surface. The excess energy results in vibrational excitation of multiple bonds and the possibility for multiple bond breaking. Here we focus on bond breaking that results in the formation of a roaming H_2_, which is the first step toward the production of H_3_^+^. When tracing H_2_ formation in ethanol we noticed three different pathways; the main pathway is from the two α-hydrogens, which accounts for about 65% from all the observed H_2_ trajectories. The second pathway is initiated with the migration of an α-hydrogen to the β-carbon, followed by the formation of H_2_ from one α- and one β-hydrogen. This second pathway was observed in about 29% of the H_2_ trajectories. The remaining 6% of H_2_ molecules were formed from two β-hydrogens. Interestingly, this pathway was initiated with the migration of an α-hydrogen to the β-carbon prior to the ejection of neutral H_2_, i.e. α-hydrogen migration preceded the ejection of two β-hydrogens. Videos showing these three H_2_ formation pathways are provided as Supplementary Videos 3‒5.

## Discussion

In summary, we have studied the reaction pathways and ultrafast dynamics associated with the formation of H_3_^+^ from a series of alcohol molecules with varying primary carbon chain lengths and molecular structures. Ab initio electronic structure calculations and molecular dynamics simulations, together with in-depth experimental data analysis allow us to refine our understanding of the relatively unknown mechanisms that lead to hydrogen molecule formation, roaming, and H_3_^+^ formation. The results presented in this Communication confirm the prevalence of roaming H_2_ molecule mechanisms in the formation of H_3_^+^. From our findings from methanol, ethanol, and 1-propanol, it is evident that the elongation of C–H bonds and narrowing of the H–C–H angle are the primary initial steps in the formation of neutral roaming H_2._ Our key experimental finding, supported by ab initio calculations, indicates that the yield of H_3_^+^ decreases as the carbon chain length increases from methanol to 1-propanol. The clear implication is that a mere increase in the number of hydrogen atoms does not necessarily result in increased H_3_^+^ yield as H_3_^+^ formation pathways are defined by unique features of the molecular structure, such as the prevalence of α-hydrogen atoms. Furthermore, through experimental evaluation of isotopically substituted ethanol, CH_3_CD_2_OD and CD_3_CH_2_OH, we unraveled four distinct H_3_^+^ formation pathways for alcohol molecules with long primary carbon chain lengths. Observation of D_3_^+^ from ethanol isotopologue CD_3_CH_2_OH, and formation of H_3_^+^ from *tert*-butanol, together with our previous results from acetone^14^ point to the existence of a lower-yield pathway exclusively involving hydrogen atoms in a terminal methyl group.

The neutral H_2_ roaming chemical reactions studied here provide insights into the exotic and hitherto unknown chemical processes occurring in our universe. Specifically, the combined experimental and theoretical work presented here explores the existence of roaming reactions occurring in ionic species, which have not been widely studied thus far. Based on time-resolved measurements following ultrafast double ionization of small alcohols and confirmed by molecular dynamics simulations, we observe that these H_2_ roaming chemical reactions occur in the 100–260 fs timescale. Given that roaming fragments spend time in relatively flat regions of the potential energy surface, the dynamics of these reactions are much slower than those of direct unimolecular dissociation. These reactions take place following double ionization (27.0–30.5 eV) and involve hydrogen atoms, which are very light. Direct dissociation should be expected to be faster than 20 fs, especially because of the Coulombic repulsion within the small molecule. The measured reaction times (100–260 fs) are an order of magnitude slower than what one would expect if the reaction pathways did not involve roaming.

Details learned by studying the unimolecular photodissociation reactions, or “half collisions”^53,54^, presented in this study enhance our understanding of H_3_^+^ reaction mechanisms (i.e. reactive collisions). Most importantly, we find the H_2_ roaming molecule behaves as a Brønsted–Lowry base, abstracting a proton to form H_3_^+^. In turn, H_3_^+^ can behave as a Brønsted–Lowry acid, donating a proton in interstellar reactive collisions. These fundamental findings reveal an important aspect about molecular hydrogen formation from organic compounds under high-energy conditions, the chemistry mediated by the roaming hydrogen molecules, and improve our understanding of the chemical reactions that resulted in the organic compounds that likely led to life in the universe.

## Methods

### Experimental setup

A detailed description of experimental setups, parameter settings, and intensity calibration methods utilized in acquiring TOF-MS, pump-probe transients, and CTOF measurements can be found in our previous work^14^. Some salient information relevant to reproducing the data presented in this study are briefly stated below.

Both TOF-MS and pump-probe transients were acquired using a 1-kHz CPA Ti:sapphire laser system delivering 1-mJ pulses with a transform-limited duration (full width at half maximum in intensity) of 38 ± 2 fs and a Wiley-McLaren mass spectrometer. In order to maintain high reliability and reproducibility of data across all molecules, we took great care to maintain crucial experimental parameters, such as laser pulse duration, pulse energy, beam pointing, sample density, and detector bias as close as possible among acquisitions. Occurrence of asymmetric ion yields in our previously published^14^ mass spectra were eliminated by employing a circular slit with a diameter of 12.5 mm. Even though this change may have caused an increased volume effect, we did not observe any significant influence on the presented results. All liquid organic samples (with percent purity better than 99.9%) were first dehydrated for more than 24 h using an ample amount of 4-Å molecular sieve desiccants and outgassed using several iterations of freeze-pump-thaw cycles. During all the measurements carried out, the sample gas pressure inside the mass spectrometer was kept at (3.5 ± 0.5) × 10^−7^ Torr (corrected for Bayard–Alpert ion gauge sensitivity for the specific gas sample being measured), approximately three orders of magnitude higher than the typical base pressure. The intensity of the pump beam was kept at 2.0 × 10^14^ W cm^−2^ and the probe beam’s intensity was set to 1.0 × 10^14^ W cm^−2^. Polarization of the pump beam was kept parallel to the TOF axis and that of the probe was set perpendicular to the pump. No long-lived doubly-charged parent ion, which is the essential precursor state needed for H_3_^+^ formation, was observed due to ionization by the probe beam. During TOF measurements, all ions produced within the same laser shot were detected using a chevron micro-channel plate (MCP) detector assembly coupled to a 500 MHz, 2 Gsa s^−1^ digital oscilloscope. The ion detection efficiency of the detector plates was not taken into account since in this configuration, the MCP detection efficiency was shown to exhibit a minimal *m*/*z* dependence^62,63^. Each data point of a given pump-probe transient (e.g. Fig. 4) is an average value of more than 3 × 10^5^ laser shots. The measured signal has an uncertainty lower than 5%.

CTOF data were acquired using a Cold Target Recoil Ion Momentum Spectroscopy (COLTRIMS)^64,65^ setup and a CPA Ti:sapphire laser system, known as PULSAR, operating at 10 kHz repetition rate delivering energies up to 2 mJ per pulse. The pulse duration was measured to be approximately 35 fs during the measurements presented in Fig. 2 and reduced to 23 fs for data shown in Fig. 3. High-purity liquid samples were introduced to the UHV chamber subsequent to thorough outgassing. The laser beam was focused to a peak intensity up to 2.0 × 10^14^ W cm^−2^ (3.0 × 10^14^ W cm^−2^ for CTOF measurements presented in Fig. 3) and directed into a skimmed molecular beam created by a supersonic gas jet, producing doubly-charged parent precursors. The polarization of the incident laser beam was set parallel to the TOF axis. The event rate recorded by the detector was kept below 1 event/shot to reduce random coincidence events. In order to obtain statistically significant results, each acquisition lasted more than 10^8^ laser shots. The analytical expression used for the evaluation of total H_3_^+^ production branching ratios, derived in Supplementary Note 1, is given by

2 $$F_T\left( 3 \right) = \mathop {\sum}\limits_j {F_2\left( {3,j} \right)} = \frac{{\mathop {\sum}\limits_j {M\prime \left( {3,j} \right)} }}{\varepsilon \mathop {\sum}\limits_l {M_2\left( l \right)} + \mathop {\sum}\limits_{k \le j} {M\prime \left( {k,j} \right)}}{.}$$

Observing in some previous published works that the C^4+^ yield at *m*/*z* = 3 of the mass spectrum had been erroneously assigned to H_3_^+^ due to degeneracy in *m*/*z*, we took extra caution to confirm that there is no contribution from C^4+^ to the ion yield at *m*/*z* = 3 in our data. In all acquired mass spectra (Supplementary Figs. 1‒5), we observed no ions at *m*/*z* = 4, which can be assigned to C^3+^, above our detection threshold, an essential precursor for formation of C^4+^. Thus, we conclude any contribution from C^4+^ yield to our data is insignificant.

### Ab initio calculations and simulations

The structural rearrangements following ionization were assessed by performing geometry optimization calculations for the neutral and the doubly-charged structures of methanol and ethanol at the CCSD/aug-cc-PVDZ level of theory. Mulliken population analysis was carried out at the EOM-CCSD/cc-PVQZ level of theory for both the neutral and doubly-charged electronic configurations of the optimized neutral structures of each alcohol. All CCSD geometry optimizations were carried out using the Molpro 2012.1 software package^66–68^ while the EOM-CCSD property calculations were performed using GAMESS^69,70^. Ab initio molecular dynamics for the dissociation of doubly-charged ethanol were carried out using the CASSCF method employing 12 electrons in 12 orbitals as an active space. The 6-31G** basis set was used. We have also carried out dynamics simulations using QCISD with the basis set 6-311G**. The validity of QCISD was benchmarked for methanol and compared with our previously reported CASSCF results^14^. The trajectories’ initial positions and momenta were sampled from the vibrational Wigner distribution for the neutral ground state computed in the harmonic approximation at each of the aforementioned levels of theory. The dynamics were integrated up to 300 fs while utilizing the velocity Verlet integrator with a time step of 0.5 fs. A total of 2000 trajectories was computed for each method. CASSCF trajectories were calculated using a development version of TeraChem^71–75^ while QCISD calculations were carried out using Molpro 2012.1^76–78^.

## Electronic supplementary material

Supplementary Information

Description of Additional Supplementary Files

Supplementary Movie 1

Supplementary Movie 2

Supplementary Movie 3

Supplementary Movie 4

Supplementary Movie 5

## Data Availability

The data that support the findings of this study are available within the Supplementary Information and upon reasonable request from the corresponding author.
