# Supplementary material for: H2 roaming chemistry and the formation of H3+ from organic molecules in strong laser fields
Source: Nat Commun. 2018 Dec 5;9:5186. doi: 10.1038/s41467-018-07577-0 (PMC6281587; doi:10.1038/s41467-018-07577-0)
Supplement: Supplementary file 1 — Supplementary Information [file 41467_2018_7577_MOESM1_ESM.pdf]

Supplementary Information

H_2 roaming chemistry and the formation of H_3^+ from organic molecules in strong laser fields

Ekanayake *et al.*

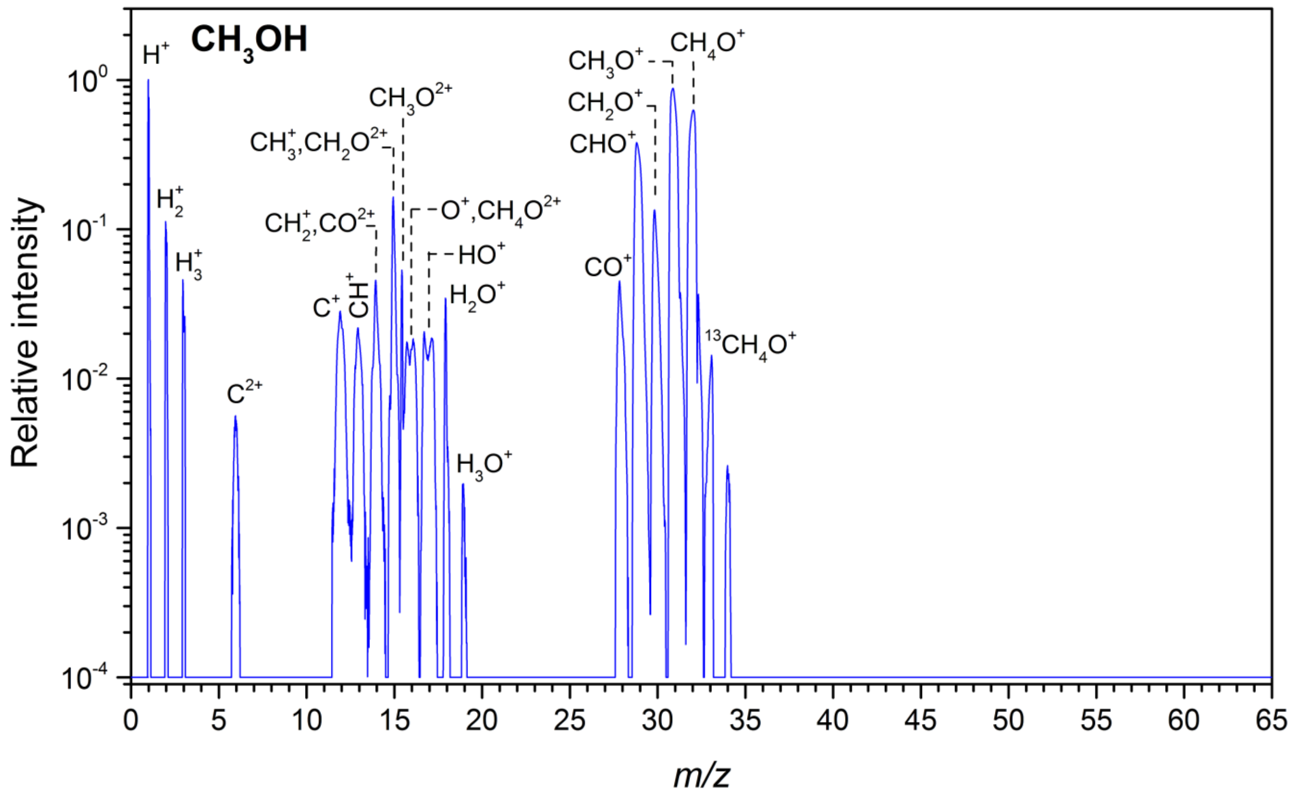

Supplementary Figure 1. CH_3OH mass-spectrum. Time-of-flight mass spectrum for dissociative ionization of dehydrated CH_3OH (methanol) in a linearly polarized laser focus of 2.0×10^{14} W cm^{-2}. Note that in the mass spectrum, no C^{3+} yield at $m/z = 4$ was observed.

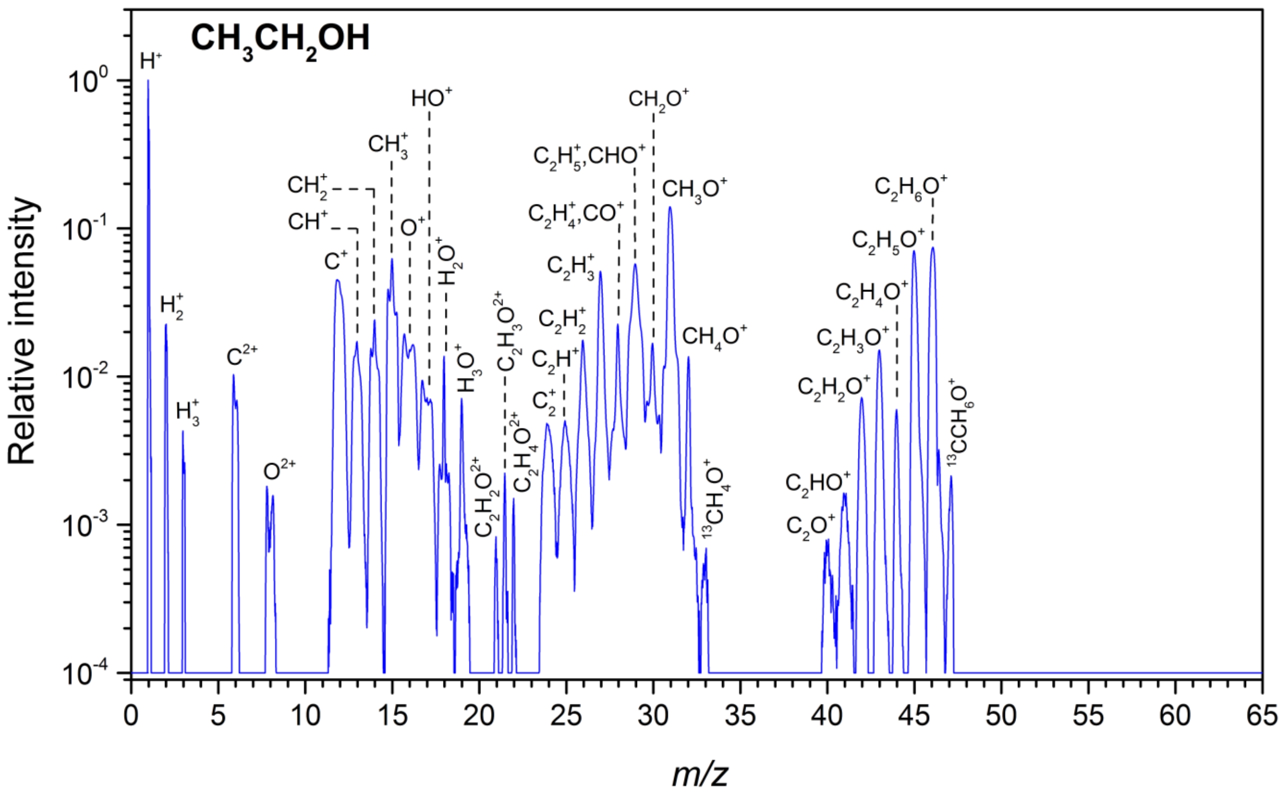

Supplementary Figure 2. CH_3CH_2OH mass spectrum. Time-of-flight mass spectrum for dissociative ionization of dehydrated CH_3CH_2OH (ethanol) in a linearly polarized laser focus of 2.0×10^{14} W cm^{-2}. Note that in the mass spectrum, no C^{3+} yield at $m/z = 4$ was observed.

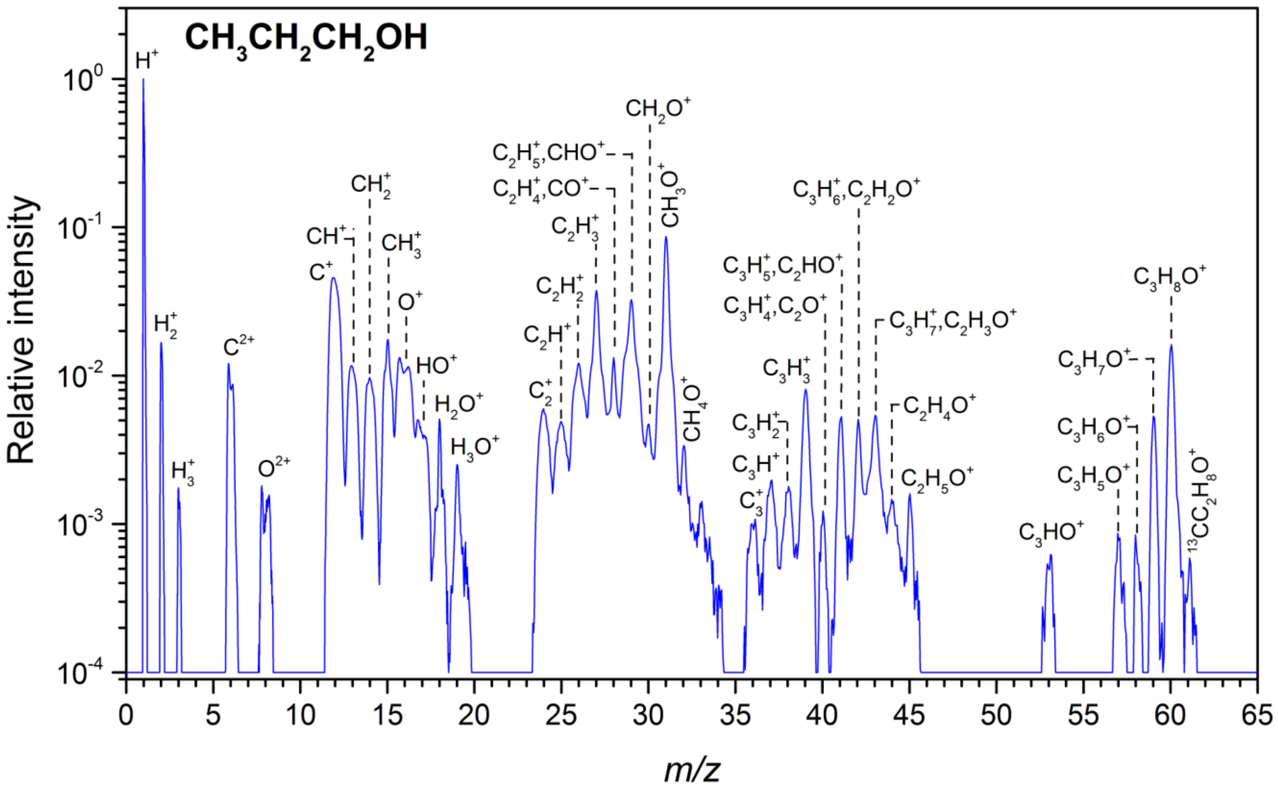

Supplementary Figure 3. $CH_3CH_2CH_2OH$ mass spectrum. Time-of-flight mass spectrum for dissociative ionization of dehydrated $CH_3CH_2CH_2OH$ (1-propanol) in a linearly polarized laser focus of 2.0×10^{14} W cm^{-2}. Note that in the mass spectrum, no C^{3+} yield at m/z = 4 was observed.

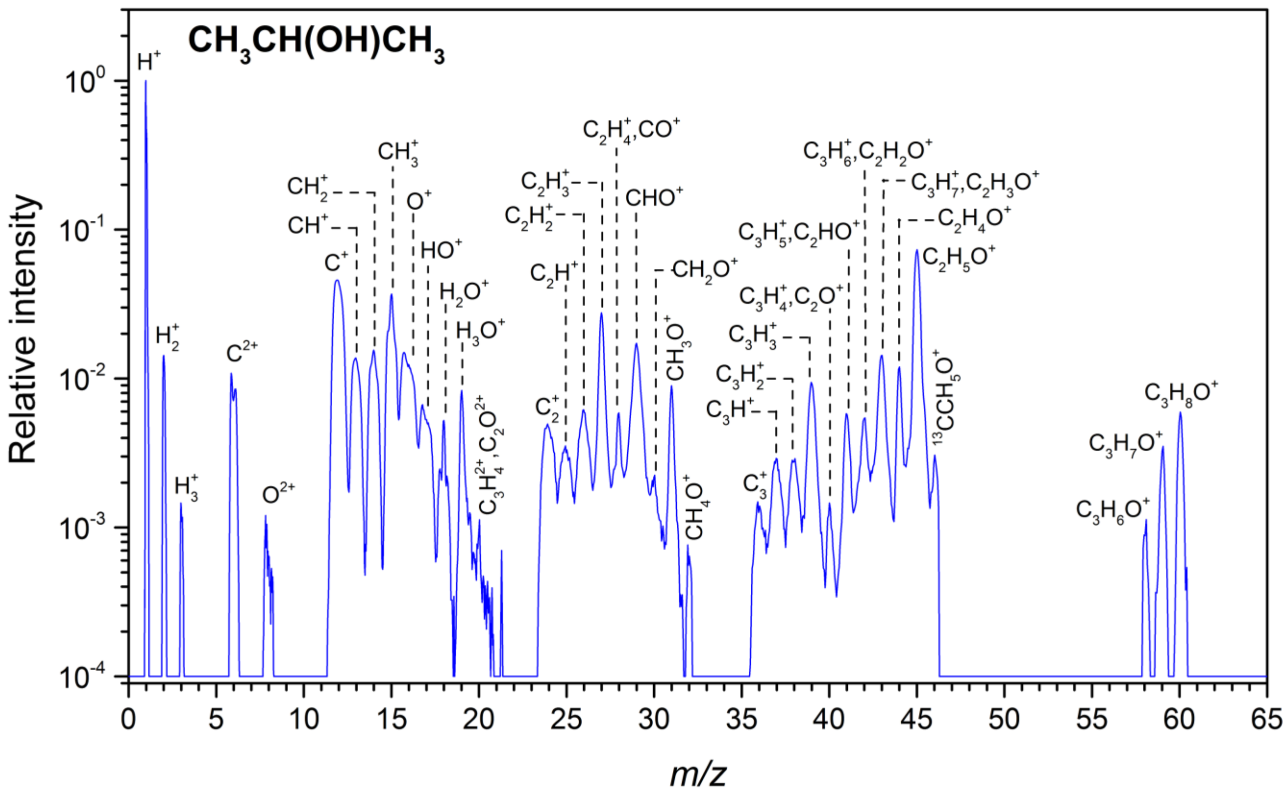

Supplementary Figure 4. $CH_3CH(OH)CH_3$ mass spectrum. Time-of-flight mass spectrum for dissociative ionization of dehydrated $CH_3CH(OH)CH_3$ (2-propanol) in a linearly polarized laser focus of 2.0×10^{14} W cm^{-2}. Note that in the mass spectrum, no C^{3+} yield at m/z = 4 was observed.

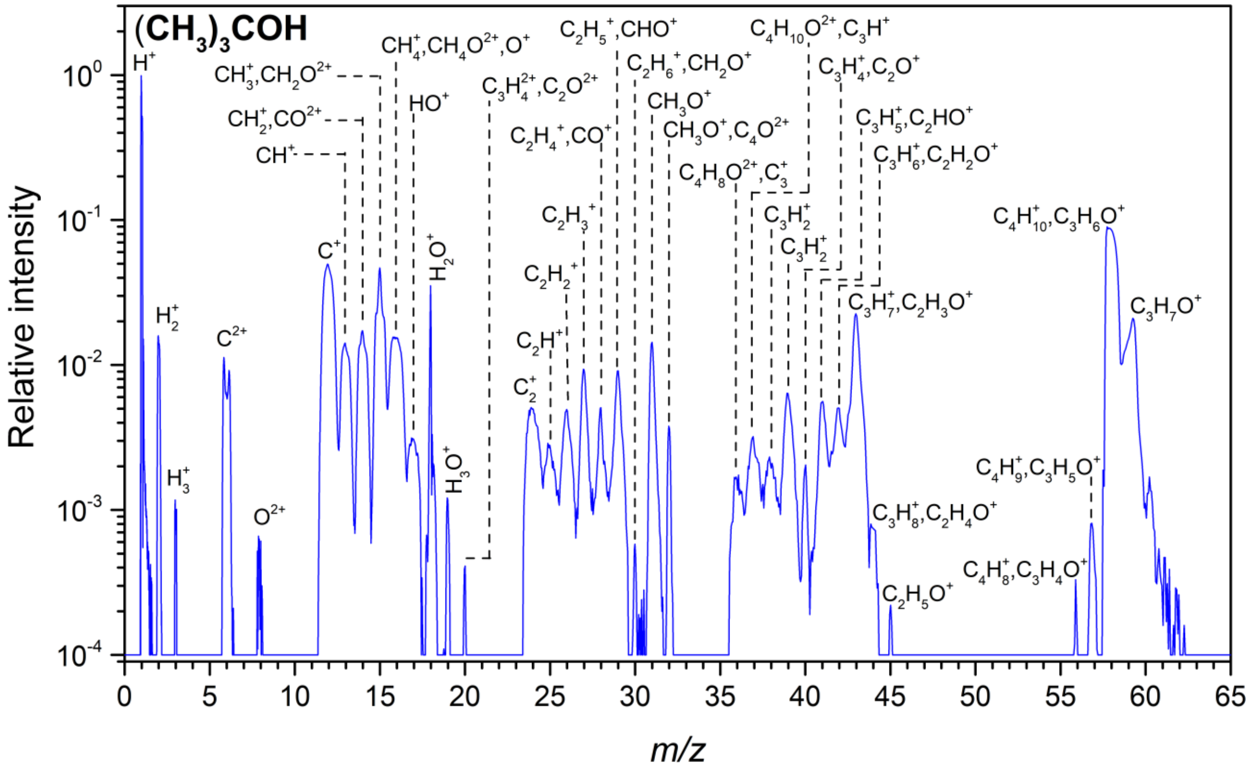

Supplementary Figure 5. $(CH_3)_3COH$ mass spectrum. Time-of-flight mass spectrum for dissociative ionization of dehydrated $(CH_3)_3COH$ (*tert*-butanol) in a linearly polarized laser focus of 2.0×10^{14} W cm^{-2}. Note that in the mass spectrum, no C^{3+} yield at m/z = 4 was observed. No prominent ion peaks beyond m/z = 65 were observed except a peak at m/z = 68 with a relative intensity of ~10^{-3}.

Supplementary Note 1 – Normalization of TOF and CTOF data for H_3^+ production

Here we describe methods for normalizing measurements of different molecules in order to compare the production rate of a specific product – H_3^+ from alcohol molecules in our case.

Interactions causing single and double ionization of target molecules produce single ions $S_q(i)$ and ion pairs $C_q(i, j)$, where q is the number of electrons ionized. The ions are usually distinguished by their m/q, but for simplicity we use integer labeling of the peaks in the TOF spectrum. Note that single ions, specifically dications, can be produced following double ionization – we include the cation and dication parent molecule among the single ions.

It is common to define the fragmentation pattern, or the branching ratios of each parent ion, as the fraction of a specific ion, or ion pair, relative to the sum of all ions originating from the same parent molecular ion. For single ionization, denoted by the subscript 1,

$$F_1(i) = \frac{S_1(i)}{\sum_k S_1(k)}. \quad (1)$$

Similarly, the branching ratios in the case of double ionization (denoted by the subscript 2) are given by

$$F_2(i) = \frac{S_2(i)}{\sum_l S_2(l) + \sum_{k \le j} C_2(k, j)},$$

for dications, and

$$F_2(i, j) = \frac{C_2(i, j)}{\sum_l S_2(l) + \sum_{k \le j} C_2(k, j)}, \quad (2)$$

for ion pairs.

Note that the denominator in Eq. (1) and Eq. (2) is the total number of singly or doubly-ionized parent molecules, respectively.

To simplify the problem, we assume that triple ionization is negligible, a fact that can be verified from the data. Moreover, it is helpful to focus on a specific example, so we choose the total H_3^+ production as our test case. For this fragment the equations above reduce to a single equation as the coincidence data suggests that H_3^+ is formed (predominantly) from the parent dication in ion-pair

breakup channels, i.e. H_3^+ + m_x^+ + m_r [where $m_r = m - (3 + m_x)$], therefore $S_1(3) = 0$ and $S_2(3) = 0$. Then, we can write the total H_3^+ production branching ratio as

$$F_T\left(3\right) = \sum_j F_2(3, j) = \frac{\sum_j C_2(3, j)}{\sum_l S_2(l) + \sum_{k \le j} C_2(k, j)}, \tag{3}$$

where $F_T\left(3\right)$ is defined by summing all the branching ratios of the relevant ion-pair channels. Our goal is to evaluate $F_T\left(3\right)$ for one molecule and compare it with the value determined for another molecule.

Next we consider the measured number of single ions $M(i)$ and ion-pairs $M(i, j)$ out of the total number of single ions $S(i)$ and ion-pairs $C(i, j)$ produced by the interaction. These quantities are related by a set of coupled equations that can be written as

$$M(i) = \varepsilon_i S_1(i) + \varepsilon_i S_2(i) + \varepsilon_i \sum_j C_2(i, j), \tag{4}$$

where ε_i is the detection efficiency of the ith fragment, the first and second terms are single ions from single and double ionization, respectively, and the third term is due to fragments from ion pairs (i.e. the detection of either both ions without preserving their coincidence information or only one ion out of the pair caused by the less than unity detection efficiency). Similarly, the measured number of ion-ion coincidences is given by

$$M(i, j) = \varepsilon_i \varepsilon_j C_2(i, j) + \tau \varepsilon_i \varepsilon_j \left[S_1(i) + S_2(i)\right]\left[S_1(j) + S_2(j)\right], \tag{5}$$

where the first term is due to true ion-pairs, while the second is due to random (also known as "false") coincidences associated with the random-coincidence rate coefficient, τ. Random-coincidence events can be also due to a single ion and an ion-pair, two ion pairs, etc. produced in the same pulse. These higher order contributions are much less likely and for simplicity they are not shown in Eq. (5), however, they are all subtracted by our random-pair subtraction algorithm. These random coincidences can be subtracted from Eq. (5) before proceeding. To that end, we generate a large set of purely random ion-pairs by pairing ions generated in different laser pulses (taking advantage of the data recorded event by event). That data set is scaled to the measured data using a purely random

coincidence ion pair, for example CH_3OH^+ + OH^+ coincidences in methanol, and then subtracted (see, sub section iii.). Using the resulting “random-coincidence free” spectra we evaluate

$$M'(i,j) = \varepsilon_i \varepsilon_j C_2(i,j). \tag{6}$$

For our example, H_3^+ production, Eqs. (4) and (6) simplify to

$$M(3) \simeq \varepsilon_3 \sum_j C_2(3,j), \tag{7}$$

$$M'(3,j) \simeq \varepsilon_3 \varepsilon_j C_2(3,j), \tag{8}$$

as we can neglect the $S_1(3)$ [see, our recent work[1] where we used the high KER of the H_3^+ fragment to exclude breakup from the cation] and the $S_2(3)$ terms. Assuming the same detection efficiency for all ions the equations above simplify further to

$$M(3) \simeq \varepsilon \sum_j C_2(3,j), \tag{9}$$

$$M'(3,j) \simeq \varepsilon^2 C_2(3,j). \tag{10}$$

i. TOF normalization

Now, we define the normalized yield of H_3^+, also called the “fractional H_3^+ yield” in the main paper, as the ratio of the measured number of H_3^+ ions and the sum over all ions measured in a TOF spectrum, that is

$$\frac{M(3)}{\sum_k M(k)} \simeq \frac{\varepsilon \sum_j C_2(3,j)}{\varepsilon \sum_m S_1(m) + \varepsilon \sum_l S_2(l) + \varepsilon \sum_{k \le j} C_2(k,j)}, \tag{11}$$

which simplifies to

$$\frac{M(3)}{\sum_i M(i)} \simeq \frac{\sum_j C_2(3,j)}{\sum_m S_1(m) + \sum_l S_2(l) + \sum_{k \le j} C_2(k,j)}. \tag{12}$$

Recall that the number of ions produced by photo-ionization (or a similar linear process) can be written as

$$S_1(m) = \int_0^t y_1(m)dt = \int_0^t \sigma_1 F_1(m) N_T n_p dt, \tag{13}$$

where σ_1 is the single ionization cross section, $F_1(m)$ is the fraction of a specific ion produced by single ionization, N_T is the target number density and n_p is the number of photons per second. It is important to note that $\sum_m F_1(m) = 1$ and $\sum_l F_2(l) + \sum_{k \le j} F_2(k, j) = 1$.

Assuming that the target density is not fluctuating too much, the expression above can be simplified to

$$S_1(m) = \sigma_1 F_1(m) \overline{N_T} \int_0^t n_p dt = \sigma_1 F_1(m) \overline{N_T} N_p, \tag{14}$$

where $\overline{N_T}$ is the average target density, and N_p is the total number of photons in the measurement. A similar expression can be written for the double ionization leading either to single ions, i.e. $A^{2+} + B$, or an $A^+ + B^+$ ion pair.

Substituting the equation above (and the similar equation for double ionization) in Eq. (12) yields

$$\frac{M(3)}{\sum_k M(k)} \simeq \frac{\sum_j \sigma_2 F_2(3, j) \overline{N_T} N_p}{\sum_m \sigma_1 F_1(m) \overline{N_T} N_p + \sum_l \sigma_2 F_2(l) \overline{N_T} N_p + \sum_{k \le j} \sigma_2 F_2(k, j) \overline{N_T} N_p}. \tag{15}$$

Note that, as expected, the target density and the number of photons (or the number of laser pulses) cancel out, however, the single and double photoionization cross sections do not. Dividing the numerator and denominator in the equation above by σ_2 yields

$$\frac{M(3)}{\sum_k M(k)} \simeq \frac{\sum_j F_2(3, j)}{\frac{\sigma_1}{\sigma_2} \sum_m F_1(m) + \sum_l F_2(l) + \sum_{k \le j} F_2(k, j)}, \tag{16}$$

which can be further simplified to

$$\frac{M(3)}{\sum_k M(k)} \simeq \frac{\sum_j F_2(3, j)}{\frac{\sigma_1}{\sigma_2} + 1}. \tag{17}$$

by using $\sum_m F_1(m)=1$ and $\sum_l F_2(l)+\sum_{k,j} F_2(k,j)=1$.

It is important to note that comparing the H_3^+ production between two molecules using the normalization procedure detailed above yields

$$\frac{M(3)/\sum_k M(k)}{M''(3)/\sum_{k''} M''(k'')} \simeq \frac{\left(\frac{\sigma''_1}{\sigma''_2}+1\right)}{\left(\frac{\sigma_1}{\sigma_2}+1\right)} \frac{\sum_j F_2(3,j)}{\sum_{j''} F_2''(3,j'')}, \tag{18}$$

and not the desired $\sum_j F_2(3,j)/\sum_j F_2''(3,j)$ ratio.

Note that σ_1/σ_2 can be a very large number because it is much easier to ionize one electron than two. More importantly, σ_1 and σ_2 typically differ significantly from one molecule to another, especially in a strong field for which ionization is known to depend strongly on I_p – and I_p is different for the alcohols we have studied[2]. Therefore, this normalization approach may not accurately provide the desired ratio needed in order to compare between molecules. Moreover, the assumption that H_3^+ is not a product of the cation may not be valid for all molecules.

ii. CTOF normalization

To circumvent the issue with the normalization method described above, we employ coincidence time-of-flight (CTOF) and define the total H_3^+ branching ratio as the ratio between the sum of the H_3^+ + m_x^+ ion pairs produced and the sum over all events involving the parent dication molecule, that is

$$F_T(3) \equiv \frac{\sum_j C_2(3,j)}{\sum_l S_2(l)+\sum_{k\le j} C_2(k,j)}. \tag{19}$$

Here, we need to determine *first* the number of ion-pairs produced, $C_2(k,j)$ after subtraction of the random coincidences using Eq. (6), which can be rewritten as

$$C_2(k,j)=\frac{1}{\varepsilon^2} M'(k,j). \tag{20}$$

assuming the same detection efficiency, ε, for all ions. Similarly the few A^{2+} + B and AB^{2+} yields, $S_2(i)$, need to be determined by using Eq. (4), which can be rewritten as

$$S_2(i) = \frac{1}{\varepsilon} M_2(i), \tag{21}$$

assuming no overlap with singly charged fragment ions in TOF, i.e. no $S_1(i)$ contribution to the specific peak of interest. Note that there are no "lost-fragments" contributions[3,4] as we neglected triple ionization and higher. The evaluation of $S_2(i)$ and $C_2(i, j)$ may require solving the set of coupled Eqs. (4) and (5) if the simplifications above do not hold. Once $S_2(i)$ and $C_2(i, j)$ are known, the normalized yield of H_3^+ defined as

$$F_T(3) \equiv \frac{\sum_j C_2(3, j)}{\sum_l S_2(l) + \sum_{k \le j} C_2(k, j)}, \tag{22}$$

can be related to the branching ratios by substituting Eq. (14) into the expression above, leading to

$$F_T(3) = \frac{\sum_j \sigma_2 F_2(3, j) \overline{N_T} N_p}{\sum_l \sigma_2 F_2(l) \overline{N_T} N_p + \sum_{k \le j} \sigma_2 F_2(k, j) \overline{N_T} N_p}, \tag{23}$$

which simplifies to

$$F_T(3) = \frac{\sum_j F_2(3, j)}{\sum_l F_2(l) + \sum_{k \le j} F_2(k, j)}, \tag{24}$$

as the average target density, $\overline{N_T}$, integrated number of photons, N_p, as well as the double ionization cross section, σ_2, cancel out. Finally, taking advantage of $\sum_l F_2(l) + \sum_{k \le j} F_2(k, j) = 1$ the expression above reduces to the desired normalized branching ratio,

$$F_T(3) = \sum_j F_2(3, j), \tag{25}$$

which can be compared to other molecules. Explicitly, the total H_3^+ production branching ratio can be directly compared for two molecules

$$\frac{F_T(3)}{F_T''(3)} = \frac{\sum_j F_2(3, j)}{\sum_{j''} F_2''(3, j'')}, \tag{26}$$

but we have to keep in mind that *first* we have to evaluate all $S_2(i)$ and $C_2(i, j)$ either by solving the coupled Eqs. (4) and (5), or by subtracting the random coincidences first and taking advantage of the fact that the doubly-charged dications in our case do not overlap singly charged ions.

Finally, using Eqs. (3), (20), and (21), the explicit expression for the total H_3^+ production branching ratio as a function of the measured yields is given by

$$F_T(3) = \sum_j F_2(3, j) = \frac{\sum_j M'(3, j)}{\varepsilon \sum_l M_2(l) + \sum_{k \leq j} M'(k, j)}. \tag{27}$$

iii. Evaluating true coincidence events of ion pairs

The CTOF ion-pair spectra consist of both true and random (also referred to as false) coincidences. The random coincidences are due to the accidental detection of two ions from different molecules produced in the same laser pulse and pass all the conditions we impose on our ion pairs. The main source of these random events are two ions produced by single ionization, as those have a higher rate.

The spectrum of the random coincidences can be generated by pairing ions recorded for different laser pulses in our data set, which is recorded event-by-event. In Supplementary Fig. 6 we show an example of the raw CTOF data of ethanol together with the respective random coincidences scaled to match, for example the purely random H_3O^+ + CH_3O^+ coincidence peak. The true coincidence spectra are generated by subtracting the scaled random coincidence spectra from the raw spectra and are shown in panels (c) and (f).

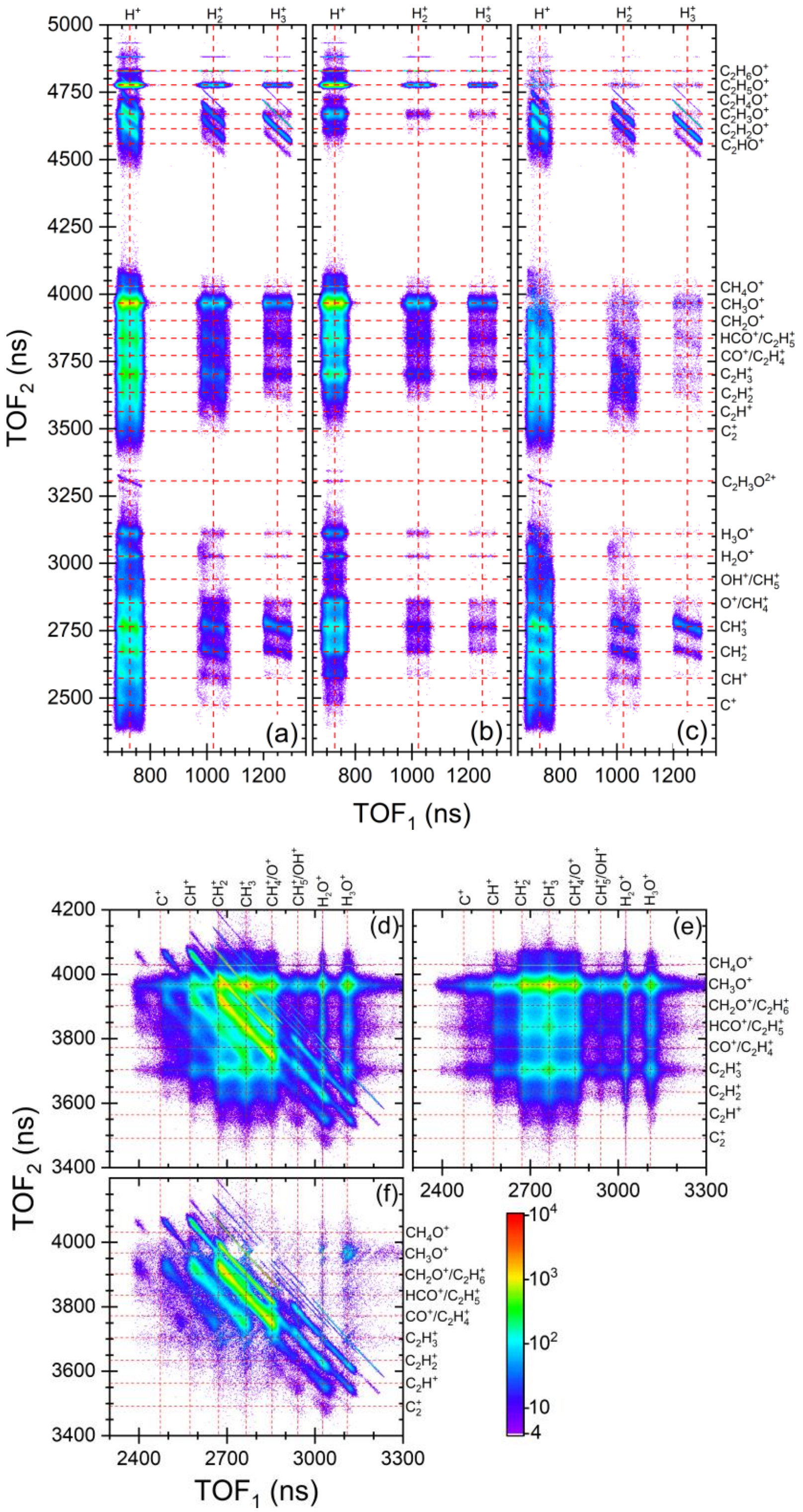

Supplementary Figure 6. Coincidence time-of-flight spectra of ethanol. The raw data are shown in panels (a) and (d) for smaller and larger *m/q* first hits, respectively. Panels (b) and (e) show the respective random coincidences generated from the same data set (see text). The respective true coincidence spectra are shown in panels (c) and (f).

Supplementary Note 2 – TOF and CTOF analysis for H_3^+ production

i. CTOF and TOF comparison

Supplementary Table 1. Data corresponding to total H_3^+ branching ratios (CTOF) and H_3^+ production (TOF) shown in Fig. 2. The TOF data was normalized to match the CTOF branching ratio for methanol.

Molecule	H_3^+ branching ratio	[H_3^+]	[H_3^+]/[all ions]
methanol	0.142 ± 0.014	0.142 ± 0.016	0.142 ± 0.017
ethanol	0.0288 ± 0.0029	0.044 ± 0.005	0.051 ± 0.006
1-propanol	$0.00250^{+0.00250}_{-0.00027}$	0.024 ± 0.003	0.023 ± 0.003
2-propanol	Not measured	0.021 ± 0.002	0.020 ± 0.002
tert-butanol	Not measured	0.019 ± 0.002	0.015 ± 0.002

The errors for H_3^+ branching ratios are detailed in the next sub section. The errors indicated for [H_3^+] yield include both systematic and statistical errors. Errors for [H_3^+]/[all ions] are calculated through error propagation.

ii. CTOF branching ratios

As discussed in Supplementary Note 1, to compare the total H_3^+ production rate we need to evaluate the branching ratio of H_3^+ production as well as many other breakup channels of the dication. In addition, we need to determine the detection efficiency so that the AB^{2+} + N breakup can be added to $AB^+ + CD^+$ + N channels (where N denotes neutral fragments), as their detection efficiencies are different (ε for the former and ε^2 for the latter, assuming the same detection efficiency for all ions).

The detection efficiency was determined using the methanol and ethanol CTOF measurements, for which the H_3^+ production from the monocation is negligible (as suggested by the kinetic-energy distribution of these fragments)[1].

The errors in the branching ratios include statistical errors, errors due to subtraction of random pairs (i.e. false coincidences), and the estimated error due to losses and scaling of the generated random events.

Methanol (CH_3OH)

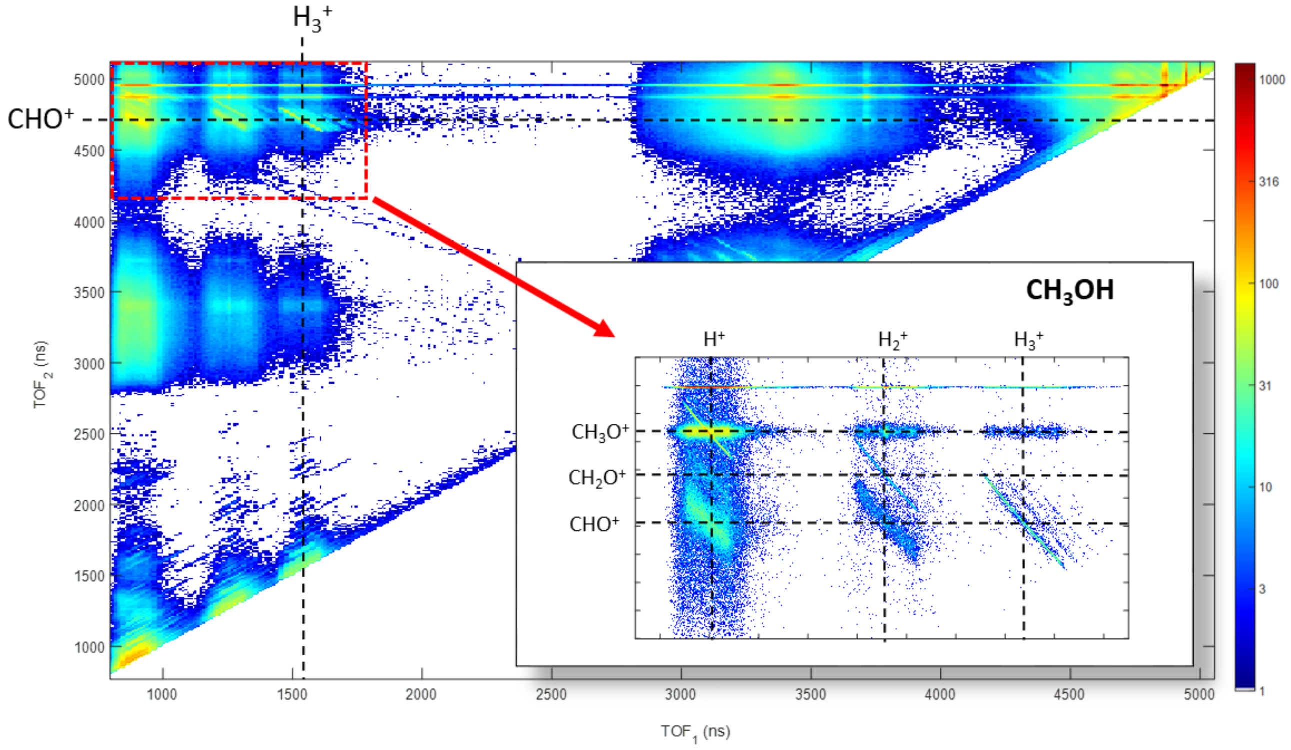

Supplementary Figure 7. Truncated coincidence time-of-flight spectrum of methanol. The CTOF spectrum from dissociative ionization of methanol in a linearly polarized laser focus of 2.0×10^{14} W cm^{-2}. The magnified view of H^+, H_2^+, and H_3^+ formation channels is given in the inset. The logarithmic color scale indicates the number of events recorded.

Detection efficiency: $\varepsilon = 0.278 \pm 0.028$

Branching ratios:

$H_3^+ + CHO^+$ 0.142 ± 0.014

Ethanol (CH_3CH_2OH)

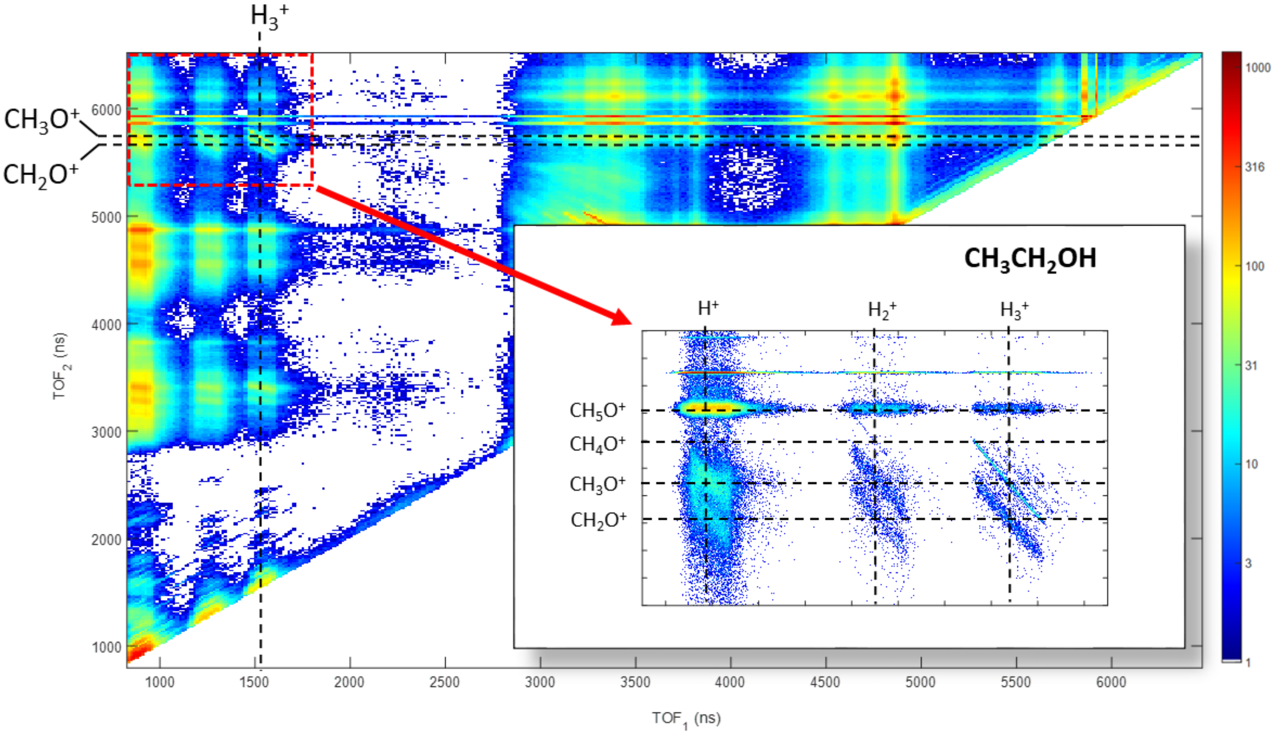

Supplementary Figure 8. Truncated coincidence time-of-flight spectrum of ethanol. The CTOF spectrum from dissociative ionization of ethanol in a linearly polarized laser focus of 2.0×10^{14} W cm^{-2}. The magnified view of H^+, H_2^+, and H_3^+ formation channels is given in the inset. The logarithmic color scale indicates the number of events recorded.

Detection efficiency: $\varepsilon = 0.230 \pm 0.023$

Branching ratios:

Sum of H_3^+ channels	0.0288 ± 0.0029	
$H_3^+ + CH_3^+ + CO$	0.0108	± 0.0011
$H_3^+ + C_2H_3O^+$	0.00784	± 0.00079
$H_3^+ + C_2H_2O^+ + H$	0.00633	± 0.00064
$H_3^+ + CH_2^+ + HCO$	0.00272	± 0.00028
$H_3^+ + C_2HO^+ + 2H/H_2$	0.00104	± 0.00011

1-Propanol ($CH_3CH_2CH_2OH$)

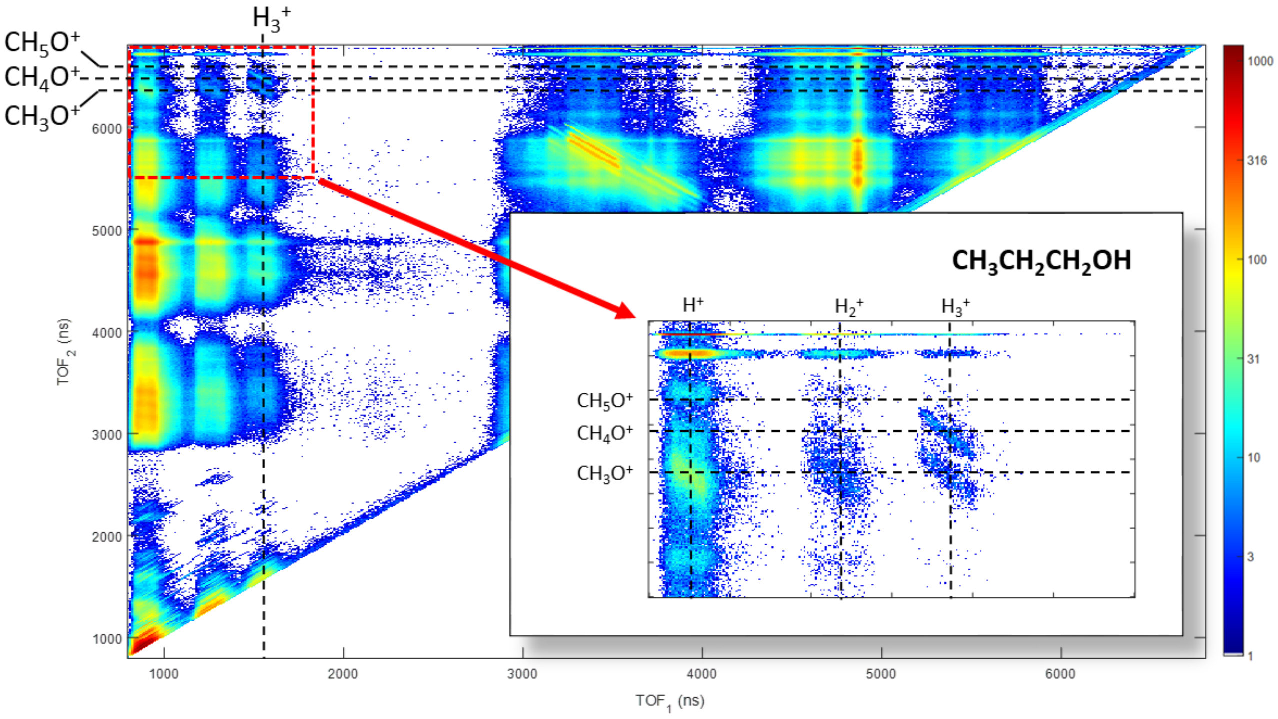

Supplementary Figure 9. Truncated coincidence time-of-flight spectrum of 1-propanol. The CTOF spectrum from dissociative ionization of 1-propanol in a linearly polarized laser focus of 2.0×10^{14} W cm^{-2}. The magnified view of H^+, H_2^+, and H_3^+ formation channels is given in the inset. The logarithmic color scale indicates the number of events recorded.

Detection efficiency: $\varepsilon = 0.254 \pm 0.025$

Branching ratios:

Sum of H_3^+ channels	$0.00250\,^{+0.00250}_{-0.00027}$	
H_3^+ + [$C_3H_3^+$ + H_2O or $C_3H_2^+$ + H_3O]	0.0008910	± 0.0000973
H_3^+ + $C_3H_3O^+$ + 2H/H_2	0.0006491	± 0.0000673
H_3^+ + C_3HO^+ + 4H	0.0004833	± 0.0000507
H_3^+ + $C_2H_3^+$ + CH_2O	0.0001378	± 0.0000647
H_3^+ + $C_3H_2O^+$ + 3H	0.0001250	± 0.0000149
H_3^+ + CHO^+ + C_2H_4	0.0001169	± 0.0000540
H_3^+ + $C_3H_4O^+$ + H	0.0000689	± 0.0000099
H_3^+ + $C_3H_5O^+$	0.0000295	± 0.0000052

No single dications were identified in the 1-propanol TOF spectrum (see Supplementary Fig. 3). As a result, the detection efficiency plays no role in evaluating the branching ratios of this dication. Note that the detection efficiency evaluated from the propanol run is much lower than for the other runs, suggesting possible losses of H_3^+ + ion (+ neutral) events or the production of H_3^+ from the mono-cation of propanol. We have estimated the losses in the main H_3^+ + ion channel, in order to place an

upper limit on the total H_3^+ production branching ratio, which may be somewhat higher than the 0.0025 reported above. At most, the losses are about 50%, therefore the upper limit of the “Sum of H_3^+ channels” is 0.005, which is still a factor of 5.5 smaller than in ethanol. Therefore, we report this branching ratio as 0.0025 with a positive error estimated to be 0.0025 and a negative error of 0.00027. We should note that the positive error is dominated by the losses and is a maximum error, while the negative error is at the one sigma level and includes the same error sources as for the methanol and ethanol, listed before. Another consequence of this error analysis is that it supports the assertion that some of the H_3^+ is formed from the 1-propanol monocation and not just the dication as is the case for methanol and ethanol.

Supplementary Note 3 – *Ab initio* electronic structure calculations

A comparison of the neutral and doubly-charged minima of methanol and ethanol is shown in Supplementary Fig.10 and was carried out at the CCSD/aug-cc-PVDZ level of theory. It is worth noting that diffuse basis functions were required to obtain the doubly-charged minimum, especially for ethanol, where the C−H bonds were elongated. Similar stable structures were observed for methanol with larger (aug-cc-PVTZ and aug-cc-PVQZ) basis sets. However, a stable doubly-charged structure was not found for ethanol when the larger aug-cc-PVTZ and aug-cc-PVQZ bases were employed. During optimizations with these sets the two elongated C−H bonds were broken forming a separate H_2 molecule. Our past dynamic simulations of methanol and these optimizations both suggest that the potential energy surface is quite flat in this region, and therefore the presence or absence of a true minimum is of little consequence. Whether or not a true minimum exists, these structures represent an important point along the path to H_2 formation, and we continue to investigate it as such. Despite significant effort, no stable doubly-charged minimum structures were identified for 1- or 2-propanol. Alpha cleavage occurred during optimization of these systems. Note also that the optimized doubly-charged structures presented are not the global minima on the dication potential energy surfaces. Lower energy structures are observed with the H atom of the alcohol group rotated 180 degrees into an eclipsed conformation. However, in our prior molecular dynamics study, such rotation was not observed. Thus, we focus here on the local minima closest to the staggered global neutral minimum energy structure.

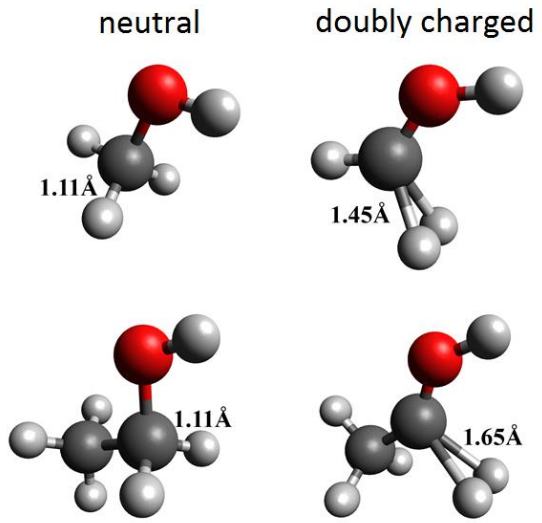

Supplementary Figure 10. Neutral and doubly-charged structure minima of methanol and ethanol along with the bond lengths of the C−H bonds that are involved in the roaming H_2 formation mechanism.

All (*x*, *y*, *z*) positions are in Angstroms.

Methanol CCSD/aug-cc-pVDZ structure:

	x	*y*	*z*
C	0.0000000195	-0.0131768233	-0.7343922430
H	-0.0000071776	1.0230451559	-1.1041231549
H	0.9012636481	-0.5256519967	-1.1187662006
H	-0.9012569859	-0.5256640351	-1.1187656406
O	0.0000000164	0.0642150004	0.6960184904
H	0.0000000220	-0.8340172099	1.0447990281

Methanol^{2+} CCSD/aug-cc-pVDZ structure:

C	0.1189100179	0.0000430246	0.5905496163
H	1.0313154368	0.0001752224	1.2430077346
H	-1.0531236166	0.4383414673	1.3285827728
H	-1.0530053232	-0.4386474885	1.3285737727
O	0.0290167368	-0.0000620036	-0.6135424060
H	-0.8027571896	0.0006023057	-1.1983973794

Ethanol CCSD/aug-cc-pVDZ structure:

C	0.5823320567	0.0000006934	0.0068561207
H	1.2316304512	0.8963327894	0.0401338985
H	1.2316318110	-0.8963302897	0.0401359596
O	-0.3266327451	0.0000013928	1.1199442120
H	0.1891694455	-0.0000207052	1.9340413694
C	-0.2532427341	-0.0000009016	-1.2680477372
H	0.4036223760	0.0000294370	-2.1551061844
H	-0.8964028113	-0.8950767849	-1.3038375078
H	-0.8964450418	0.8950459252	-1.3038078383

Ethanol^{2+} CCSD/aug-cc-pVDZ structure:

C	0.2215308728	0.0004025632	0.0444691126
H	1.8243587117	0.4100894434	0.1128925228
H	1.8225276032	-0.4109498723	0.1112697220
O	-0.2382919152	-0.0000507140	1.1546073811
H	0.2056842234	-0.0008655943	2.0593889450
C	-0.1658509121	0.0000191134	-1.3506316157
H	0.2684836791	-0.9097264325	-1.8358787519
H	-1.2797579380	0.0050777582	-1.3693431502
H	0.2776945931	0.9021548395	-1.8411003929

The neutral structure minima of the four alcohols were also obtained at the CCSD/cc-pVQZ level of theory. At the neutral structure minima, the density matrix and Mulliken population analysis were evaluated for neutral and doubly-charged electronic configurations at the EOM-CCSD/cc-pVQZ level of theory. This allows us to probe the change in electron density under the assumption of an instantaneous (much faster than nuclear rearrangement) double ionization. Atomic charges for both electronic configurations are depicted in Supplementary Fig. 11.

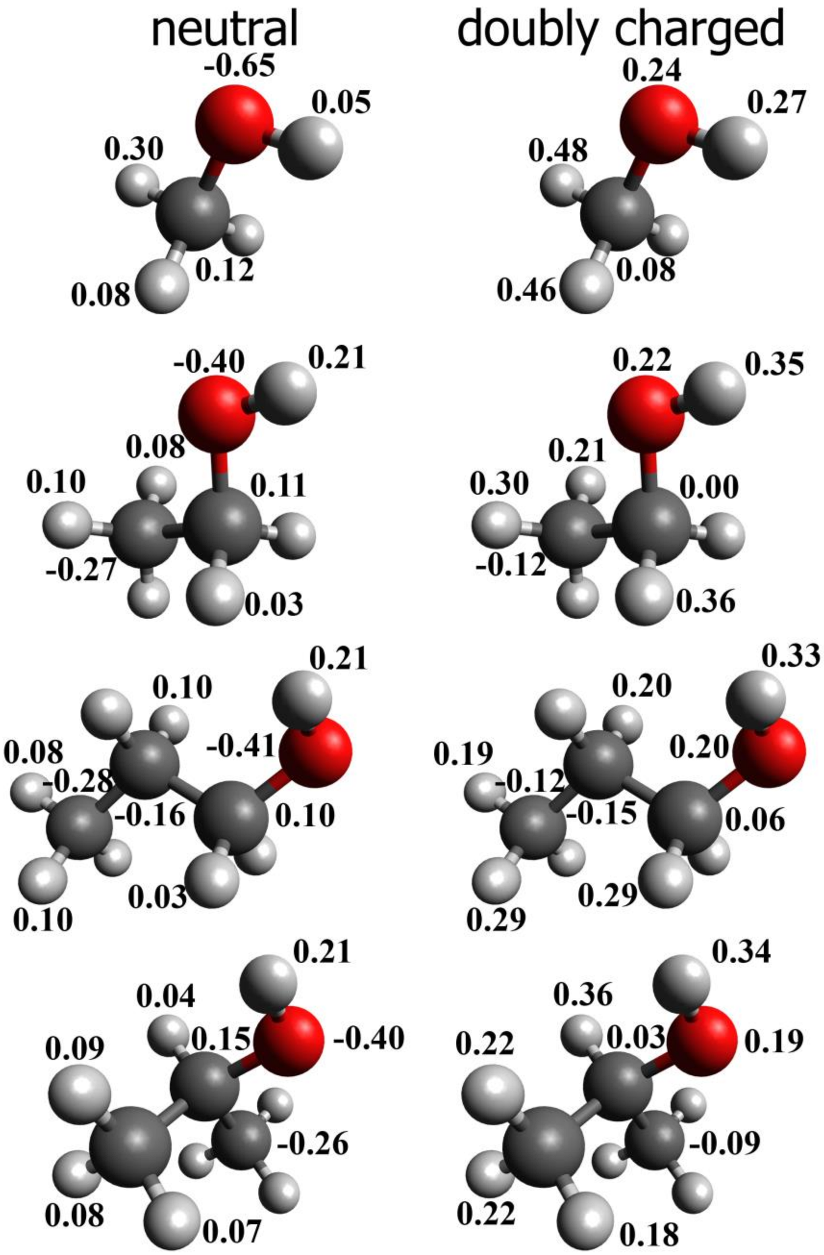

Supplementary Figure 11. Mulliken atomic charges of methanol, ethanol, 1-propanol, and 2-propanol (top to bottom) for the neutral and doubly-charged electronic configurations. Atomic charges on atoms that are equivalent by symmetry are not shown.

All (*x*, *y*, *z*) positions are in Angstroms.

	x	*y*	*z*
**Methanol CCSD/cc-pVQZ structure:**			
C	0.0000000694	-0.0132012215	-0.7243533803
H	-0.0000229410	1.0070988969	-1.0967123079
H	0.8882245538	-0.5188207406	-1.1076907792
H	-0.8882033680	-0.5188591048	-1.1076888181
O	0.0000000565	0.0637854542	0.6871491326
H	0.0000000316	-0.8245980541	1.0363955536
**Ethanol CCSD/cc-pVQZ structure:**			
C	0.5741964862	0.0000010542	0.0097229849
H	1.2176776373	0.8835033672	0.0396304336
H	1.2176781238	-0.8835006756	0.0396329899
O	-0.3230829773	0.0000021071	1.1082310436
H	0.1851242443	-0.0000310863	1.9168350897
C	-0.2487682980	-0.0000013115	-1.2572665551
H	0.3990380445	0.0000459135	-2.1327591430
H	-0.8844826432	-0.8825184153	-1.2942669471
H	-0.8845484542	0.8824705151	-1.2942206836
**1-Propanol CCSD/cc-pVQZ structure:**			
C	0.4930178502	-0.0249404907	0.5382662111
H	1.1626047753	0.8315684531	0.4691154338
H	1.1080542798	-0.9263216560	0.4475173667
O	-0.0966631449	0.0532492160	1.8227097378
H	-0.6722992419	-0.7018955823	1.9329465254
C	-0.5248461452	0.0212641769	-0.5870540632
H	-1.2040986537	-0.8299502239	-0.4893294041
H	-1.1290408202	0.9220009357	-0.4739439700
C	0.1425395256	-0.0052700172	-1.9562967112
H	0.7423506609	-0.9067848773	-2.0842995761
H	-0.5948891807	0.0197494086	-2.7564111722
H	0.8024100820	0.8529971896	-2.0847756021
**2-Propanol CCSD/cc-pVQZ structure:**			
C	-0.0159115345	0.3695240977	0.0066974781
H	0.0097040168	1.4647090165	0.0083600582
O	1.3066595780	-0.1324317989	-0.1328388204
H	1.8149854532	0.1399616563	0.6299247250
C	-0.6488330620	-0.1128198868	1.3007613359
H	-1.6623032797	0.2744247409	1.4038547201
H	-0.6873525459	-1.2016692465	1.3137227831
H	-0.0735326642	0.2210004244	2.1653994638
C	-0.7812832287	-0.1001014046	-1.2121787195
H	-1.8010911859	0.2819061196	-1.1947719236
H	-0.2938692287	0.2446418896	-2.1216589733
H	-0.8162028895	-1.1889754161	-1.2316258925

When comparing the neutral and the doubly-charged configurations, one can clearly see that the largest increase in the positive charge on the H atoms within a molecule is associated with the ones that are on the α-C. This change is larger for the two H atoms that are *gauche* relative to the H atom of the alcohol group compared to the *anti*-H in the case of methanol. Moreover, the H atoms of methanol exhibited larger increase in positive charge than those of ethanol. This charge thus correlates with the experimentally observed H_3^+ yields.

All CCSD geometry optimizations were carried out using Molpro 2012.1[5-7] software package while the EOM-CCSD part was calculated using GAMESS[8,9].

Supplementary Note 4 – *New H_3^+ formation pathways*

Here we discuss the features and important information pertaining to the proper identification of H_3^+ formation pathways from CTOF spectra shown in Fig. 3 of the main text. As an example, we use the CTOF spectrum from the dissociative ionization of CH_3CD_2OD (Fig. 3(a) of main text), which is also shown as Supplementary Fig.12.

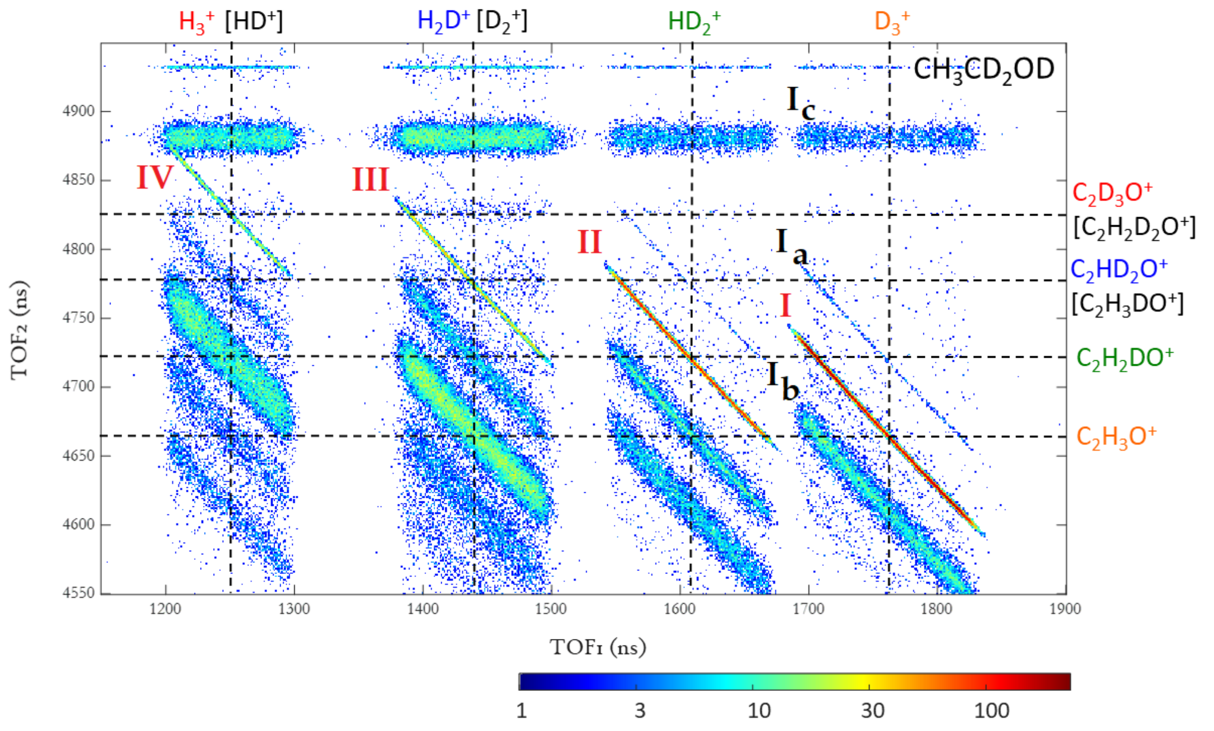

Supplementary Figure 12. H_3^+ formation from ethanol. Truncated coincidence time-of-flight map focused only on H_3^+ production in two-body channels from dissociative ionization of CH_3CD_2OD in a linearly polarized laser pulse centered about 790 nm, 23-fs long with a peak intensity of 3.0×10^{14} W cm^{-2}. The logarithmic color scale depicts the number of ion pairs recorded.

Among several coincidence channels visible on the CTOF map shown in Supplementary Fig. 12 (contour regions at the intersections of vertical and horizontal dashed lines with an approximate slope of −1), our attention primarily focused on four channels, which are sharp thin contour regions labeled as I, II, III, and IV. These channels correspond to true two-body breakup ion pairs related to H_3^+ formation from the ethanol dication, which conserve momentum during photodissociation. For instance, the channel labeled by I represents $CH_3CD_2OD^{2+} \rightarrow C_2H_3O^+ + D_3^+$ and channel II represents $CH_3CD_2OD_2^+ \rightarrow C_2H_2DO^+ + HD_2^+$. Due to mass-to-charge degeneracy in H_2D^+ (m/z = 4) and D_2^+ (m/z = 4) ions, the two-body breakup channels corresponding to the formation of these two ions, $CH_3CD_2OD2^+ \rightarrow C_2HD_2O^+ + H_2D^+$ and $CH_3CD_2OD2^+ \rightarrow C_2H_3DO^+ + D_2^+$ respectively, become indistinguishable in the CTOF map and are labeled as III. Similarly, the channel labeled by IV represents two degenerate two-body breakup channels corresponding to the formation of H_3^+ and HD^+.

Apart from the above four two-body breakup ion-pair channels, there are many other channels visible on the CTOF map. The channel represented by the label I_a is due to isotopic impurity in the sample. This is typically due to the natural occurrence of ^{13}C at the 2.2% level in the sample (since the probability of a given C atom being ^{13}C is 1.1% due to natural abundance). Wide and dispersed contour regions on the CTOF map correspond to breakup channels involving more than two breakup partners, typically involving an ion pair and a third fragment that is predominantly neutral (uncharged). For instance, the channel labeled by I_b corresponds to the three-body breakup $CH_3CD_2OD2^+ \rightarrow C_2H_2O^+ + D_3^+ + H$. The spread of the channel width is defined by the momentum absorbed by the third fragment during the photodissociation process. Horizontal contour regions, e.g. channel I_c, represent false (random) coincidences, where the paired ions arise from two separate parent dications in the focal region produced by the same laser pulse. These false events contain no useful information in quantitative analysis of CTOF spectra and can be removed (see Supplementary Note 1 (iii) for more information).

Supplementary Note 5 – *Ab initio* molecular dynamics simulations

Assessment of the validity of QCISD *ab initio* molecular dynamics for H_2 and H_3^+ formation is carried out on methanol and compared to our previous CASSCF results[1] as summarized in Supplementary Table 2. We notice that QCISD was incapable of predicting the formation of H_2^+ due to the open shell nature of that fragment. On the other hand, H_2 and H_3^+ formation yields were higher than CASSCF. This bias towards closed shell fragments provides a higher probability to observe the H_3^+ formation in ethanol which wasn't observed using CASSCF.

Supplementary Table 2. Percentage of hydrogen species (summed over all channels) ejected from doubly-charged methanol that are observed using CASSCF and QCISD *ab initio* molecular dynamics simulations.

	CASSCF % Yield	QCISD % Yield
H^+ formation	48.3	43.9
H_2^+ formation	23.4	0
H_2 formation	18.8	40.3
H_3^+ formation	4.0	5.4

Supplementary References

1. Ekanayake, N. *et al.* Mechanisms and time-resolved dynamics for trihydrogen cation (H_3^+) formation from organic molecules in strong laser fields. *Sci. Rep.* **7**, 4703 (2017).
2. Linusson, P. *et al.* Double photoionization of alcohol molecules. *Phys. Rev. A* **80**, 1–6 (2009).
3. Ben-Itzhak, I., Carnes, K. D., Johnson, D. T., Norris, P. J., & Weaver, O. L. Fragmentation of CH_4 caused by fast-proton impact. *Phys. Rev. A* **47**, 3748 (1993).
4. Ben-Itzhak, I., Carnes, K. D., Johnson, D. T., Norris, P. J. & Weaver, O. L. Velocity dependence of ionization and fragmentation of methane caused by fast-proton impact. *Phys. Rev. A* **49**, 881 (1994).
5. Werner, H.-J.; Knowles, P. J.; Knizia, G.; Manby, F. R.; Schütz, M., Molpro: a general-purpose quantum chemistry program package. *Wiley Interdiscip. Rev. Comput. Mol. Sci.* 2012, ***2***, 242-253.
6. *MOLPRO, version 2012.1, a package of ab initio programs* H.-J. Werner, P. J. K., G. Knizia, F. R. Manby, M. Schütz, P. Celani, W. Györffy, D. Kats, T. Korona, R. Lindh, A. Mitrushenkov, G. Rauhut, K. R. Shamasundar, T. B. Adler, R. D. Amos, A. Bernhardsson, A. Berning, D. L. Cooper, M. J. O. Deegan, A. J. Dobbyn, F. Eckert, E. Goll, C. Hampel, A. Hesselmann, G. Hetzer, T. Hrenar, G. Jansen, C. Köppl, Y. Liu, A. W. Lloyd, R. A. Mata, A. J. May, S. J. McNicholas, W. Meyer, M. E. Mura, A. Nicklaß, D. P. O'Neill, P. Palmieri, D. Peng, K. Pflüger, R. Pitzer, M. Reiher, T. Shiozaki, H. Stoll, A. J. Stone, R. Tarroni, T. Thorsteinsson, M. Wang. see *http://www.molpro.net*.

7. Hampel, C.; Peterson, K. A.; Werner, H.-J., A comparison of the efficiency and accuracy of the quadratic configuration interaction (QCISD), coupled cluster (CCSD), and Brueckner coupled cluster (BCCD) methods. *Chem. Phys. Lett.* ***190***, 1-12 (1992).
8. Piecuch, P.; Kucharski, S. A.; Kowalski, K.; Musiał, M., Efficient computer implementation of the renormalized coupled-cluster methods: The R-CCSD[T], R-CCSD(T), CR-CCSD[T], and CR-CCSD(T) approaches. *Comp. Phys. Commun.* ***149***, 71-96 (2002).
9. Włoch, M.; Gour, J. R.; Kowalski, K.; Piecuch, P., Extension of renormalized coupled-cluster methods including triple excitations to excited electronic states of open-shell molecules. *J. Chem. Phys.* ***122***, 214107 (2005).
