# Supplementary material for: H2 roaming chemistry and the formation of H3+ from organic molecules in strong laser fields
Source: Nat Commun. 2018 Dec 5;9:5186. doi: 10.1038/s41467-018-07577-0 (PMC6281587; doi:10.1038/s41467-018-07577-0)
Supplement: Supplementary file 2 — Description of Additional Supplementary Files [file 41467_2018_7577_MOESM2_ESM.docx]

**Description of Additional Supplementary Files**

**File Name**: Supplementary Video 1

**Description**: H3 + Formation Trajectory 1: Ab initio molecular dynamics trajectory showing the formation of H3+ which starts with the formation of neutral H2 molecule that roams around and finally abstracts the hydroxyl proton.

**File Name**: Supplementary Video 2

**Description**: H3 + Formation Trajectory 2: Ab initio molecular dynamics trajectory showing the formation of H3 + which starts with the formation of neutral H2 molecule that roams around and finally abstracts the hydroxyl proton.

**File Name**: Supplementary Video 3

**Description:** Neutral H2 Formation Trajectory from Alpha Position: Ab initio molecular dynamics trajectory for the formation of neutral H2 molecule originating from the two α-hydrogens.

**File Name**: Supplementary Video 4

**Description**: Neutral H2 Formation Trajectory from Alpha and Beta Positions: Ab initio molecular dynamics trajectory for the formation of neutral H2 molecule in which one hydrogen originates from the α position while the other is from the β position.

**File Name**: Supplementary Video 5

**Description**: Neutral H2 Formation Trajectory from Beta Position: Ab initio molecular dynamics trajectory for the formation of neutral H2 molecule originating from the two β-hydrogens**.**
